# Plasmids of Psychrotolerant *Polaromonas* spp. Isolated From Arctic and Antarctic Glaciers – Diversity and Role in Adaptation to Polar Environments

**DOI:** 10.3389/fmicb.2018.01285

**Published:** 2018-06-18

**Authors:** Anna Ciok, Karol Budzik, Marek K. Zdanowski, Jan Gawor, Jakub Grzesiak, Przemyslaw Decewicz, Robert Gromadka, Dariusz Bartosik, Lukasz Dziewit

**Affiliations:** ^1^Department of Bacterial Genetics, Institute of Microbiology, Faculty of Biology, University of Warsaw, Warsaw, Poland; ^2^Department of Antarctic Biology, Institute of Biochemistry and Biophysics, Polish Academy of Sciences, Warsaw, Poland; ^3^Laboratory of DNA Sequencing and Oligonucleotide Synthesis, Institute of Biochemistry and Biophysics, Polish Academy of Sciences, Warsaw, Poland

**Keywords:** *Polaromonas*, plasmid, Arctic, Antarctica, glacier, bacterial adaptation, heavy metal resistance, iron–sulfur [Fe-S] cluster

## Abstract

Cold-active bacteria of the genus *Polaromonas* (class *Betaproteobacteria*) are important components of glacial microbiomes. In this study, extrachromosomal replicons of 26 psychrotolerant *Polaromonas* strains, isolated from Arctic and Antarctic glaciers, were identified, sequenced, and characterized. The plasmidome of these strains consists of 13 replicons, ranging in size from 3,378 to 101,077 bp. *In silico* sequence analyses identified the conserved backbones of these plasmids, composed of genes required for plasmid replication, stable maintenance, and conjugal transfer. Host range analysis revealed that all of the identified plasmids are narrow-host-range replicons, only able to replicate in bacteria of closely related genera (*Polaromonas* and *Variovorax*) of the *Comamonadaceae* family. Special attention was paid to the identification of plasmid auxiliary genetic information, which may contribute to the adaptation of bacteria to environmental conditions occurring in glaciers. Detailed analysis revealed the presence of genes encoding proteins potentially involved in (i) protection against reactive oxygen species, ultraviolet radiation, and low temperatures; (ii) transport and metabolism of organic compounds; (iii) transport of metal ions; and (iv) resistance to heavy metals. Some of the plasmids also carry genes required for the molecular assembly of iron–sulfur [Fe-S] clusters. Functional analysis of the predicted heavy metal resistance determinants demonstrated that their activity varies, depending on the host strain. This study provides the first molecular insight into the mobile DNA of *Polaromonas* spp. inhabiting polar glaciers. It has generated valuable data on the structure and properties of a pool of plasmids and highlighted their role in the biology of psychrotolerant *Polaromonas* strains and their adaptation to the environmental conditions of Arctic and Antarctic glaciers.

## Introduction

*Polaromonas* spp. (*Betaproteobacteria*) are Gram-negative, chemoorganotrophic bacteria (Garrity, 2005; Sizova and Panikov, 2007). Their cells are motile due to the presence of a polar flagellum, and they have gas vesicles that provide buoyancy in aquatic habitats (Walsby, 1994; Garrity, 2005). So far, the genus includes nine species, and only a few have been described in detail. These include strains of biotechnological value (e.g., nitrogen fixing *Polaromonas naphtalenivorans*, able to metabolize toxic atrazine and naphthalene) and isolates with unique properties (e.g., *Polaromonas hydrogenivorans*, which uses hydrogen and carbon dioxide as sole sources of energy and carbon during autotrophic growth) (Devers et al., 2007; Sizova and Panikov, 2007; Yagi et al., 2009; Hanson et al., 2012). Until now (April 25, 2018), 436 strains have been assigned to the genus *Polaromonas*. Based on the manual inspection of every *Polaromonas* submission deposited in the GenBank (NCBI), we found, that 32% of these bacteria have been isolated from glaciers, ice or snow in diverse geographical locations, i.e., Antarctica, Arctic, the Himalayas, and the Alps. This suggests that this genus gathers a lot of cold-loving or cold-tolerant bacteria that are well-adapted to frozen environments. However, despite the wide distribution of cold-active *Polaromonas* spp., little is known about their biology and ecology.

Currently (April 25, 2018), 2 complete genomic sequences and 22 draft genomes of *Polaromonas* spp. have been deposited in the GenBank database (Supplementary Table S1). Strain JS666 (one of two strains with complete genomic sequences) was isolated from granular activated carbon sampled at a pump-and-treat plant in Germany. Its genome [GenBank: CP000316–CP000318] is composed of a circular chromosome (5.2 Mb) and two large circular self-transmissible plasmids (plasmid 1 – 338 kb and plasmid 2 – 360 kb). Genome-based *in silico* reconstruction of the metabolic pathways of JS666 revealed the considerable potential of this strain for use in bioremediation. JS666 carries genes possibly involved in the degradation of *n*-alkanes, cyclic alkanes, cyclic alcohols, haloalkanes, and haloacids, as well as genes conferring resistance to mercury and arsenic (Mattes et al., 2008). The second strain with completely sequenced genome, *P. naphtalenivorans* CJ2, was isolated from a coal-tar-contaminated freshwater sediment sampled at South Glens Falls, NY, United States. The CJ2 genome consists of a circular chromosome (4.4 Mb) [GenBank: CP000529] and eight extrachromosomal replicons pPNAP01–pPNAP08 (ranging in size from 6.4 to 353 kb), comprising 14% of the genome [GenBank: CP000530–CP000537] (Yagi et al., 2009). Strain CJ2 carries adaptive genes enabling the degradation of various aromatic compounds including benzoate, *p*-cresol, naphthalene, salicylate, vanillate, ferulate, and 3- and 4-hydroxybenzoate (Jeon et al., 2003, 2004; Yagi et al., 2009).

Adaptive genetic information is very often associated with plasmids, which enable its dissemination *via* horizontal gene transfer. Unfortunately, very little is known about the structure, properties, and ecological role of plasmids of *Polaromonas* spp. Only 10 complete plasmid genome sequences from members of this genus are available at the GenBank database (April 25, 2018) (Benson et al., 2017) – all originating from the aforementioned strains JS666 and CJ2.

In this study, we have analyzed 26 psychrotolerant strains of *Polaromonas* spp., paying special attention to their extrachromosomal replicons. These strains were isolated from four glaciers: the Hans and Werenskiold Glaciers from Spitsbergen Island (Arctic), and the Ecology and Baranowski Glaciers from King George Island (Antarctica).

The Hans and Werenskiold Glaciers are located on the north shore of Hornsund Fiord at the south end of Spitsbergen Island (Svalbard Archipelago) in the Arctic. Hans Glacier, a grounded tidewater glacier, has a surface area of around 57 km^2^ and extends to 100 m below sea level. The maximum ice thickness is estimated to be 400 m. Hans Glacier flows into Hornsund fjord in southern Spitsbergen. Werenskiold Glacier is a land-based valley glacier next to Hans Glacier flowing from east to west. It has an area of 27.11 km^2^ with a maximum ice thickness of 235 ± 15 m. Both of these glaciers are separated from the neighboring tundra and river-lake ecosystems by tall lateral moraines and mountain ridges (Pälli et al., 2003). A large nesting site for several bird species is located in the vicinity of Hans Glacier (Jakubas et al., 2008).

The Ecology Glacier is situated on the western shore of Admiralty Bay on King George Island (South Shetland Archipelago, Antarctica). It is a rapidly receding glacier that has an extensive fore field with a developed estuary, moraine systems, and kettle lakes and is adjacent to a penguin rookery. The Baranowski Glacier is a neighboring glacier, located a further 3.5 km to the south. Ecology and Baranowski are both outlet glaciers of the main ice cap of King George Island (Bintaja, 1995; Birkenmajer, 2002).

In the course of this study we have characterized 13 novel *Polaromonas* plasmids (size range: 3,378–101,077 bp). Our analysis of this large dataset has identified genes responsible for several plasmid-encoded traits and has revealed a putative role for these replicons in host adaptation to harsh glacial environments.

## Materials and Methods

### Sample Collection and Bacterial Isolation

Ice and cryoconite material was collected from the surface of the Ecology and Baranowski Glaciers (King George Island, Antarctica) (in January 2009) and the Hans and Werenskiold Glaciers (Spitsbergen Island, Arctic) (in August 2011) at four sites along transects running from the terminus of each glacier to the snowline at the top of the ablation zone. Porous surface ice (with the wind-exposed layer) was crushed with a sterilized (70% EtOH), deionized water-washed Tonar ice auger (158 cm long, 130 mm diameter), and transferred to sterile plastic bags using sterile plastic spatulas. Cryoconite holes were drained of water and sediment using 160 ml sterile plastic syringes and the material was transported in 500 ml sterile bottles to a field laboratory for processing within 2 h. In preparation for microbiological analysis, the ice samples were melted in a refrigerator (4°C). Cryoconite material was shaken gently on a shaker (Premed, model 327) (120 rpm, 20 min, 4°C), and the suspensions were returned to the refrigerator for 10–20 min to allow large particles to settle. Aliquots of 0.1, 0.5, and 1.0 ml of the prepared samples were plated on R2A agar, then held in darkness at 4°C for 6 weeks. After this incubation period, 30 colony types, differing in terms of size, color, shape, and other characteristics, were selected per sample for further analyses.

### Bacterial Strains, Plasmids, and Culture Conditions

Twenty six *Polaromonas* strains – 11 newly isolated and 15 identified previously (Gawor et al., 2016) were analyzed in this study. Other bacterial strains used were *Agrobacterium tumefaciens* LBA288 (Hooykaas et al., 1980), *Escherichia coli* BR825, DH5α λpir, TG1, and S17-1 λpir (Simon et al., 1983; Gibson, 1984; Bullock et al., 1987; Ludtke et al., 1989), *Pseudomonas aeruginosa* PAO1161 (Bartosik et al., 2014), and *Variovorax paradoxus* EPS (Pehl et al., 2012). The *Polaromonas* strains were grown on R3A medium (modified R2A broth, containing peptone – 1.0 g/L, tryptone – 1.0 g/L, yeast extract – 1.0 g/L, beef extract – 1.0 g/L, glucose – 1.0 g/L, sodium pyruvate – 0.5 g/L, NaH_2_PO_4_ – 0.5 g/L, K_2_HPO_4_ – 1 g/L, MgSO_4_ – 0.05 g/L) at 22°C. All other strains were grown on LB (lysogeny broth) at 30°C (*A. tumefaciens* and *V. paradoxus*) or 37°C (*E. coli* and *P. aeruginosa*). The media were solidified by the addition of 1.5% (w/v) agar. Where necessary, the media were supplemented with X-gal, IPTG, and antibiotics: kanamycin (50 μg/ml for *A. tumefaciens*, *E. coli*, and *V. paradoxus* or 500 μg/ml for *P. aeruginosa*) and rifampin (50 μg/ml). Plasmids used and constructed in this study are listed in Supplementary Table S2.

### Temperature Tolerance Analysis

The temperature tolerance of *Polaromonas* strains was tested by analyzing changes in the optical density of cultures (in comparison with non-inoculated controls) grown in clear 96-well plates, using an automated microplate reader (Sunrise TECAN, Tecan Trading AG, Switzerland) as previously described (Dziewit et al., 2013c). Overnight cultures were diluted in fresh R3A medium to an initial optical density at 600 nm (OD_600_) of 0.05. Culture aliquots (in triplicate) were dispensed into wells of microplates, and these were incubated with shaking at 4, 15, 22, 30, and 37°C for 192 h. The OD_600_ of the respective cultures was measured every 48 h.

### Heavy Metal Resistance Testing

Analytical grade salts (3CdSO_4_ × 8H_2_O, CoSO_4_ × 7H_2_O, CuSO_4_, HgCl_2_, MnSO_4_ × H_2_O, NiCl_2_, ZnSO_4_ × 7H_2_O) were used in a resistance assay performed in 96-well plates, as described previously (Dziewit et al., 2013c). Triplicate cultures of each strain were challenged with a range of concentrations of those heavy metal salts. Isolates that grew in the presence of the following heavy metal ion concentrations were considered resistant: (i) 0.1 mM Hg^2+^; (ii) 1 mM Cd^2+^, Co^2+^, Cu^2+^, Ni^2+^, or Zn^2+^; and (iii) 20 mM Mn^2+^ (Nieto et al., 1987; Nies, 1999; Abou-Shanab et al., 2007). To our knowledge the minimum inhibitory concentration of Mn^2+^ that defines a resistant strain has not been precisely determined. Therefore, for the purposes of this study, a cut-off value of 20 mM was selected based on the reported tolerance of *Thiobacillus ferrooxidans* and *E. coli* (Trevors et al., 1985; Nies, 1999).

### DNA Manipulations and Introduction of Plasmid DNA Into Bacterial Cells

Plasmid DNA was isolated using a GeneMATRIX Plasmid Miniprep DNA Purification Kit (EURx, Gdansk, Poland) or a classical alkaline lysis procedure (Birnboim and Doly, 1979). Routine DNA manipulations were carried out using standard methods (Sambrook and Russell, 2001). PCRs were performed using a KAPA HiFi PCR Kit with appropriate primer pairs (Supplementary Table S3). The following thermocycle was applied using a Mastercycler (Eppendorf, Hamburg, Germany) to amplify the desired products: initial denaturation at 95°C for 3 min followed by 35 cycles of denaturation at 98°C for 20 s, annealing at 62 to 65°C (depending on the primer pair) for 15 s, extension at 72°C for 30 s/kb, and then a final extension at 72°C for 1 min/kb.

Derivatives of the plasmid vectors pABW1 (Bartosik et al., 1997) and pBBR1MCS-2 (Kovach et al., 1994) were introduced into *A. tumefaciens* by triparental mating (Bartosik et al., 2001), *E. coli* and *P. aeruginosa* by chemical transformation (Kushner, 1978; Irani and Rowe, 1997), and *V. paradoxus* by electroporation (Pehl et al., 2012).

### Plasmid Host Range Analysis

Derivatives of pABW1 carrying replication modules of *Polaromonas* plasmids were introduced into *A. tumefaciens* LBA288, *E. coli* BR825, *P. aeruginosa* PAO1161, and *V. paradoxus* EPS. The construction of these plasmids is described in Supplementary Table S2. Since the ColE1-type replication system of pABW1 (Bartosik et al., 1997) is not functional in any of these recipient strains (*E. coli* BR825 carries a mutation within the DNA polymerase I gene that prevents ColE1-type replication), maintenance of the shuttle plasmids in the tested hosts was dependent on functions encoded within the cloned regions of the analyzed *Polaromonas* plasmids. The presence of an introduced plasmid within a host strain was confirmed by electrophoretic methods.

### DNA Sequencing

The complete nucleotide sequences of the *Polaromonas* plasmids were determined in the DNA Sequencing and Oligonucleotide Synthesis Laboratory (oligo.pl) at the Institute of Biochemistry and Biophysics, Polish Academy of Sciences. The plasmids were sequenced using an Illumina MiSeq instrument in paired-end mode with a v3 chemistry kit. The obtained sequence reads were filtered for quality and assembled using Newbler v3.0 software (Roche). Final gap closure was performed by capillary sequencing of PCR amplicons using an ABI3730xl DNA Analyser (Applied Biosystems, Waltham, MA, United States). PCR products cloned in vectors pABW1 or pBBR1MCS-2 were sequenced applying the same technology. Where necessary, primer walking was employed to obtain the complete nucleotide sequence of the inserted DNA.

### Bioinformatics

The Ribosomal Database Project (RDP) (Wang et al., 2007) was used for taxonomic assignments. Phylogenetic analyses were performed using MEGA6 (Tamura et al., 2013). Plasmid sequences were manually annotated using Artemis software (Rutherford et al., 2000). Similarity searches were performed using the BLAST programs (Altschul et al., 1997) and the Conserved Domains Database (Marchler-Bauer et al., 2017) provided by the NCBI^1^, and Pfam (Finn et al., 2016). RNA sequence searches were performed using the ARAGORN v1.2.38 (Laslett and Canback, 2004) and tRNAscan-SE programs (Lowe and Chan, 2016). EC numbers were assigned using the KEGG database (Kanehisa and Goto, 2000) and UniProt Knowledgebase (UniProtKB) (Pundir et al., 2017).

### Nucleotide Sequence Accession Numbers

Nucleotide sequences of *Polaromonas* spp. 16S rRNA genes and plasmids were deposited in the GenBank (NCBI) database under the accession numbers MG098807–MG098817 (for 16S rRNA genes) and MG869615–MG869627 (for plasmids).

## Results

### Identification of Plasmids and Characterization of Their Host Strains

**FIGURE 1:**
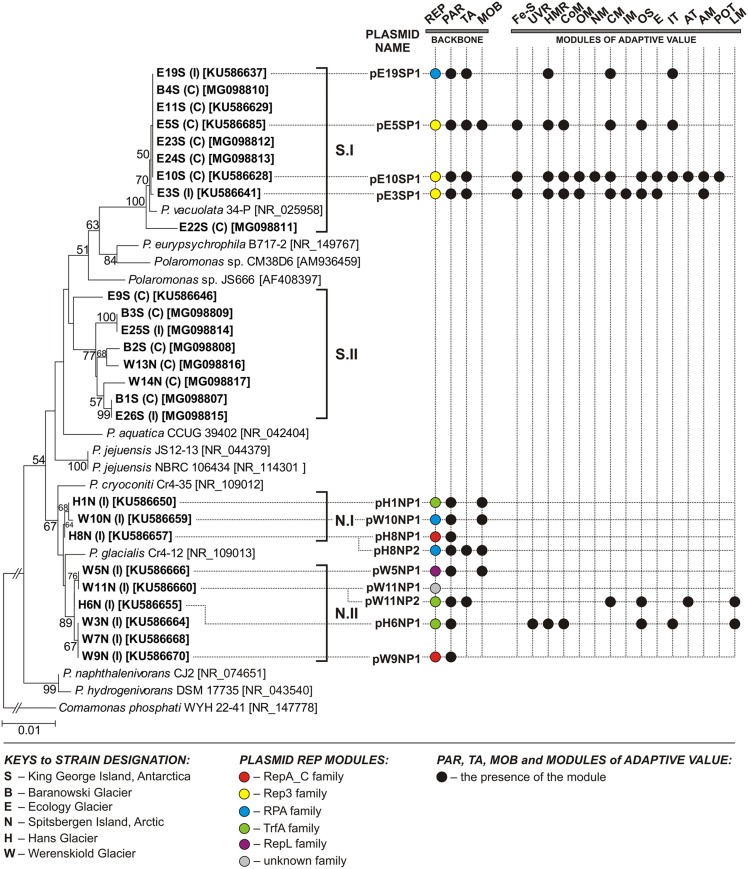
Phylogenetic tree for 16S rDNA sequences of *Polaromonas* spp. The tree was constructed by applying the neighbor-joining algorithm with Kimura corrected distances. Statistical support for the internal nodes was determined by 1000 bootstrap replicates and values of ≥50% are shown. The 16S rDNA sequence of *Comamonas phosphati* WYH 22-41 was used as an outgroup. GenBank accession numbers of the 16S rDNA sequences used for the phylogenetic analysis are given in square brackets. The scale bar represents 0.01 substitutions per nucleotide position. S.I and S.II – clusters of Antarctic strains (originating from King George Island), N.I and N.II – clusters of Arctic strains (originating from Spitsbergen Island). Strains analyzed in this study are in bold text. The source of each isolate is indicated in round brackets (I – ice, C – cryoconite material). The presence of a particular module within a given plasmid is shown by a colored dot. The following modules are indicated: AM, amino acid metabolism; AT, amino acid transport; CM, carbohydrate metabolism; CoM, coenzyme metabolism; E, energy production and metabolism; [Fe-S], [Fe-S] cluster assembly; HMR, heavy metal resistance; IM, ion metabolism; IT, ion transport; LM, lipid metabolism; MOB, conjugal transfer system; NM, nucleotide metabolism; OM, metabolism of other compounds; OS, oxidative stress response; PAR, partitioning system; POT, polyamine transport system; REP, replication system; TA, toxin–antitoxin system; UVR, UV radiation response.

Twenty six *Polaromonas* spp. strains isolated from ice and cryoconite material (collected from Arctic and Antarctic glaciers) were subjected to detailed analysis. The isolates originated from (i) the Baranowski and Ecology glaciers, located on King George Island (Antarctica) (4 and 11 strains, respectively) and (ii) the Hans and Werenskiold Glaciers on Spitsbergen Island (Arctic) (3 and 8 strains, respectively) (**Figure 1**). Fifteen of the strains (E3S, E5S, E9S, E10S, E11S, E19S, H1N, H6N, H8N, W3N, W5N, W7N, W9N, W10N, and W11N) have been described previously (Gawor et al., 2016), while the other 11 (B1S, B2S, B3S, B4S, E22S, E23S, E24S, E25S, E26S, W13N, and W14N) are new isolates. The 16S rRNA genes of the novel isolates were amplified by PCR, sequenced, and analyzed to allow taxonomic assignment and classification of the strains.

Phylogenetic analysis of the 16S rDNA sequences of all 26 isolates and 11 reference *Polaromonas* strains (from the NCBI database) showed clustering into two groups, reflecting their origin – Arctic (N cluster) or Antarctic (S cluster) (**Figure 1**). Two Arctic strains (W13N and W14N) were an exception, because they were localized in the “Antarctic cluster” on the tree. Within each main cluster, two sub-clusters were distinguished, grouping strains with related 16S rDNA sequences (**Figure 1**).

To characterize the plasmidome of *Polaromonas* spp., the aforementioned strains were screened for the presence of extrachromosomal replicons. As a result, 13 plasmids were identified – 4 in Antarctic strains (all isolated from Ecology Glacier) and 9 in Arctic strains (three isolates from Hans Glacier and four from Werenskiold Glacier). Two Arctic strains (from the latter pool), H8N and W11N, carried two plasmids each (**Figure 1** and **Table 1**).

**Table 1:** General features of *Polaromonas* spp. plasmids identified in this study.

Plasmid	Strain	Plasmid size (bp)	Family of replication protein	GC content (%)	Number of genes	% of coding region	GenBank accession number
pE3SP1	E3S	101,077	Rep_3	55.57	100	78.2	MG869617
pE5SP1	E5S	65,477	Rep_3	53.56	64	74.8	MG869618
pE10SP1	E10S	86,294	Rep_3	53.11	90	81.2	MG869615
pE19SP1	E19S	18,920	RPA	49.12	23	62.3	MG869616
pH1NP1	H1N	29,488	TrfA	55.43	24	76.0	MG869619
pH6NP1	H6N	82,545	TrfA	59.64	70	70.1	MG869620
pH8NP1	H8N	11,215	RepA_C	54.66	14	73.9	MG869621
pH8NP2	H8N	38,325	RPA	57.37	33	79.3	MG869622
pW5NP1	W5N	9,573	RepL	50.54	10	78.5	MG869626
pW9NP1	W9N	7,205	RepA_C	53.68	11	84.4	MG869627
pW10NP1	W10N	20,809	RPA	50.46	19	81.5	MG869623
pW11NP1	W11N	3,378	Unclassified	57.76	2	55.4	MG869624
pW11NP2	W11N	52,468	TrfA	58.56	55	78.9	MG869625


The plasmid-containing strains were subjected to temperature tolerance tests, and all were able to grow at temperatures ranging from 4 to 22°C (Supplementary Figure S1). Thus, these strains were classified as psychrotolerants (Morita, 1975).

The complete nucleotide sequences of the plasmids were determined and thoroughly analyzed. All replicons are circular, they range in size from 3.4 to 101.1 kb, and they have a highly varied GC content (**Table 1**). In total, 515 protein coding regions (CDSs) were distinguished in the plasmid genomes, and biological functions could be predicted for 82% of them (Supplementary Table S4). The remaining CDSs encode hypothetical proteins or proteins of unknown function.

### Plasmid Backbone Modules

**FIGURE 2:**
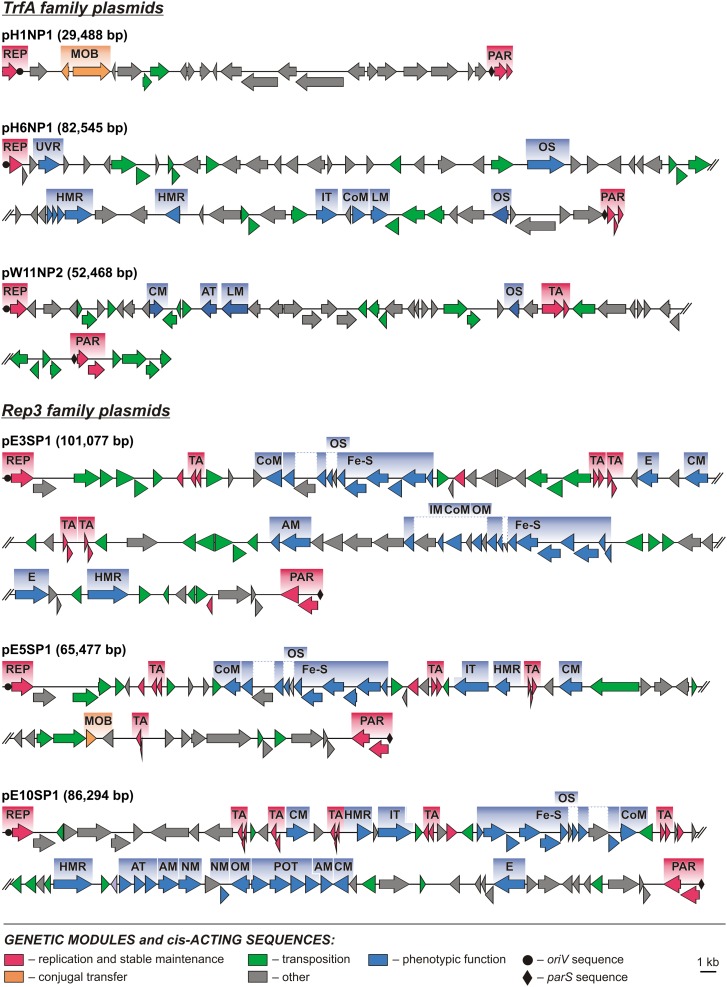
Linear maps showing the genetic structure of the circular *Polaromonas* plasmids encoding replication initiation proteins of the TrfA and Rep3 families. Arrows indicate genes and their transcriptional orientation. Predicted genetic modules are indicated by colored boxes: AM, amino acid metabolism; AT, amino acid transport; CM, carbohydrate metabolism; CoM, coenzyme metabolism; E, energy production and metabolism; [Fe-S], [Fe-S] cluster assembly; HMR, heavy metal resistance; IM, ion metabolism; IT, ion transport; LM, lipid metabolism; MOB, mobilization for conjugal transfer; NM, nucleotide metabolism; OM, metabolism of other compounds; OS, oxidative stress response; PAR, partitioning system; POT, polyamine transport system; REP, replication system; TA, toxin–antitoxin system; UVR, UV radiation response. Orphan genes encoding predicted ParAs and antitoxins of TA systems are not shown as modules. The linear maps of plasmids pE3SP1, pE5SP1, pE10SP1, pH6NP1, and pW11NP2 were divided for clarity.

**FIGURE 3:**
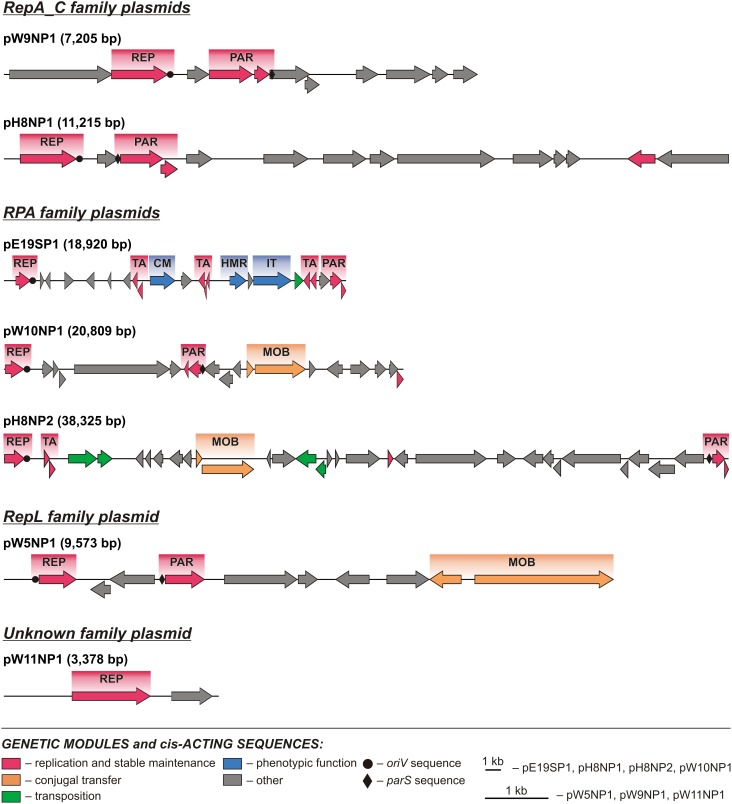
Linear maps showing the genetic structure of the circular *Polaromonas* plasmids encoding replication initiation proteins of the RepA_C, RPA, and RepL families, and unknown family plasmid pW11NP1. Arrows indicate genes and their transcriptional orientation. Predicted genetic modules are indicated by colored boxes: CM, carbohydrate metabolism; HMR, heavy metal resistance; IT, ion transport; MOB, conjugal transfer system; PAR, partitioning system; REP, replication system; TA, toxin–antitoxin system. Orphan genes encoding predicted ParAs and antitoxins of TA systems are not shown as modules.

Comparative analysis of the plasmid genome sequences enabled the identification of their conserved backbones, composed of clusters of genes involved in basic processes, such as replication (REP), plasmid stabilization (STA), and mobilization for conjugal transfer (MOB) (**Figures 2**, **3**).

#### Replication Systems and Their Host Range

Analysis of the plasmid genome sequences identified genes encoding replication initiation proteins (Rep), which were mainly classified to the Rep_3 superfamily (11 plasmids) (**Table 1** and Supplementary Table S5). The Rep_3 domain-containing proteins were further subdivided into four families: (i) RepA_C (plasmids pH8NP1 and pW9NP1), exhibiting amino acid (aa) sequence similarity to Rep of *Burkholderia pseudomallei* TSV202 plasmid 2 [GenBank: AIV73640]; (ii) Rep3 (plasmids pE3SP1, pE5SP1, and pE10SP1), with aa sequence similarity to Rep of *Rhodoferax antarcticus* DSM 24876 plasmid 2 [GenBank: APW48633]; (iii) RPA (plasmids pE19SP1, pH8NP2, and pW10NP1), with aa sequence similarity to Rep of *P. naphthalenivorans* CJ2 plasmid pPNAP06 [GenBank: ABM40236]; and (iv) TrfA (pH1NP1, pH6NP1, and pW11NP2), with aa sequence similarity to Rep of *Rhodoferax ferrireducens* T118 plasmid 1 [GenBank: ABD71980] (**Figure 1** and Supplementary Table S5).

Two *Polaromonas* spp. plasmids (pW5NP1 and pW11NP1) were found to encode different Rep proteins. The replication initiator of pW5NP1 was classified within the RepL family since it displays local sequence similarities to proteins containing a RepL-type replication domain, e.g., Tint_3268 protein of plasmid pTINT02 of *Thiomonas intermedia* K12 [GenBank: NC_014155] (64% aa identity). The Rep of pW11NP1 is unique among *Polaromonas* plasmids identified so far. This protein lacks any conserved domains, but it shares some sequence similarity with the putative replication initiators of several small plasmids, e.g., pNI10 of *Pseudomonas fulva* IF-4 [GenBank: NP_862364] and p47L of *Pseudomonas* sp. S-47 [GenBank: AAX51981].

To identify putative origins of replication (*oriV*s) within the REP modules, DNA sequences upstream and downstream of the predicted *rep* genes were searched for iteron-like repeats (i.e., putative DNA regions where Rep proteins bind) and AT-rich sequences, where replication might be initiated (del Solar et al., 1998). Likely *oriV*s were identified for the majority of the plasmids with the exception of pW11NP1, which does not contain any apparent repeated sequences. Features of the predicted *oriV*s are presented in Supplementary Table S5.

To examine the host range of the analyzed *Polaromonas* spp. plasmids, their predicted REP regions were amplified by PCR and cloned in an *E. coli*-specific mobilizable vector pABW1. This resulted in the construction of 13 shuttle plasmids (Supplementary Table S2), which were introduced into several host strains representing different classes of *Proteobacteria* (*Alpha*, *Beta*, and *Gamma*). All of these plasmids were able to replicate in *Polaromonas* spp. and six of them (pE19SP1, pH1NP1, pH6NP1, pH8NP1, pH8NP2, and pW11NP1) also functioned in *V. paradoxus* EPS, a species phylogenetically closely related to *Polaromonas* spp. However, none of the plasmids was able to replicate in strains of *Alpha*- or *Gammaproteobacteria*, which demonstrates their narrow host range.

#### Stable Maintenance Modules

Plasmids are stably maintained in bacterial cells and bacterial populations due to the action of various stabilization systems that provide (i) efficient resolution of multimeric plasmid forms (*via* multimer resolution systems, MRS), (ii) active segregation of plasmids into daughter cells during cell division (*via* partitioning systems, PAR), and (iii) post-segregational elimination of plasmid-less cells [*via* toxin–antitoxin systems (TA)] (Sengupta and Austin, 2011).

A typical PAR system consists of two genes encoding ParA and ParB proteins, which interact *in cis* with a partitioning site, *parS* (Baxter and Funnell, 2014). Twelve of the analyzed *Polaromonas* spp. plasmids carry genes encoding ParA-like proteins, with sequence similarity to partitioning ATPases assigned to the COG1192 group (Supplementary Table S6). All of these proteins contain a specific variant of the canonical Walker A motif (part of the ATP binding site) and lack a helix-turn-helix motif in their N-terminal regions (Ramakrishnan et al., 2002), which are the characteristic features of ParA proteins of class IB PAR modules (Baxter and Funnell, 2014).

In four plasmids (pE3SP1, pE5SP1, pE10SP1, and pW11NP2), *parA*-overlapping *parB* genes (assigned to the COG1475 group) were identified. The ParB proteins of pE3SP1, pE10SP1, and pE5SP1 are highly related (95% aa identity) to a putative ParB protein of *R. antarcticus* DSM 24876 plasmid 2 [GenBank: APW48631], while the ParB of pW11NP2 is similar (96% aa identity) to the ParB-like partitioning protein of pPNAP02 of *P. naphthalenivorans* CJ2 [GenBank: ABM39747]. Another *Polaromonas* plasmid, pW9NP1, encodes a ParG-type protein (equivalent to ParB), that is most similar to a hypothetical protein of *Geobacter pickeringii* G13 [GenBank: CP009788]. In all the other analyzed plasmids (pE19SP1, pH1NP1, pH6NP1, pH8NP1, pH8NP2), the genes associated with *parA* lack any sequence similarity to *parB*. Nevertheless, homologous gene pairs are conserved in other plasmid genomes (data not shown), which strongly suggests that they constitute a functional PAR module. The genetic organization of all the identified PAR *loci*, including their putative *parS* sites, is presented in Supplementary Table S6.

The *Polaromonas* spp. plasmids also contain numerous TA systems. Such systems encode two components – a toxin protein that binds a specific cellular target and an antitoxin (protein or antisense RNA), which counteracts the toxin (Leplae et al., 2011; Unterholzner et al., 2013). Twenty type II TA systems (where both the toxin and antitoxin are proteins) were identified in seven of the plasmids: pH8NP2 (1 TA *locus*), pE3SP1 (5), pE5SP1 (4), pE10SP1 (5), pE19SP1 (3), pW10NP1 (1), and pW11NP2 (1) (Supplementary Table S7). The individual components of these *loci* were classified into different TA families based on sequence homology (Supplementary Table S7). The most abundant were modules of the BrnTA family (seven *loci*) (Heaton et al., 2012) and hybrid modules encoding a ParE-like toxin and an antitoxin of the HTH_XRE-family (five *loci*) (Supplementary Table S7). Interestingly, four orphan toxin genes were also identified, encoding predicted proteins of the PemK (pH8NP1) and Doc (pE3SP1, pE5SP1, and pE10SP1) toxin families.

#### Conjugal Transfer Modules

Many plasmids may be transferred to other bacterial cells by conjugation. Amongst the most common plasmids of this type are mobilizable replicons, whose transfer depends on the presence of other self-transmissible elements in a cell. The mobilizable plasmids contain MOB modules encoding relaxase proteins, which initiate the transfer by nicking DNA at a specific origin site (*oriT*) (Smillie et al., 2010; Wawrzyniak et al., 2017).

None of the analyzed *Polaromonas* plasmids appear to carry a complete set of conjugal transfer genes, although five of them (pE5SP1, pH1NP1, pH8NP2, pW5NP1, and pW10NP1) encode relaxases. Plasmids pH8NP2 and pW10NP1 possess highly similar 1.4-kb DNA regions (76% nucleotide sequence identity), encoding a putative TraJ protein (positive regulator of *tra* gene expression) and relaxase TraI (Supplementary Table S8). Both proteins exhibit significant homology (between 41 and 58% amino acid identity) to proteins encoded by plasmid 5 of a cold-active bacterium *Burkholderia* sp. PAMC 26561, isolated from lichen on King George Island (Antarctica) [GenBank: WP_062175115 and WP_069638512, respectively]. Analysis of conserved sequence motifs of the predicted TraI of pH8NP2 and pW10NP1 allowed classification of these relaxases into the MOB_Q1_ family (Garcillan-Barcia et al., 2009). A related relaxase gene was also found in plasmid pE5SP1 (Supplementary Table S8), although it is disrupted by transposition of a Tn*3*-family element.

Two other plasmids, pH1NP1 and pW5NP1, carry homologous pairs of genes encoding a putative coupling protein (TraD) and a MobA/MobL relaxase (Supplementary Table S8). These proteins display sequence similarity to plasmid-encoded proteins of the psychrotolerant *Burkholderia* sp. PAMC 28687, isolated from Antarctica [GenBank: WP_062004363 and WP_082779226, respectively]. The presence of conserved motifs within the MobA/MobL proteins allowed their classification into the MOB_P_ family of relaxases (Garcillan-Barcia et al., 2009). None of the analyzed plasmids contain sequences similar to the *oriT*s of other well-characterized MOB modules.

### Genetic Modules of Adaptive Value

Analysis of the genetic load of the studied *Polaromonas* plasmids revealed that six of them carry genes that may directly influence the phenotype of their hosts (**Figure 1**). Some of the genes may be involved in adaptation to conditions prevailing in polar environments. Specific gene products may participate in (i) protection against reactive oxygen species (ROS), ultraviolet radiation (UV radiation), and low temperatures; (ii) transport and metabolism of organic compounds (amino acids, carbohydrates, and lipids); and (iii) transport of metal ions or (iv) resistance to heavy metals (Supplementary Table S9).

#### Protection Against Oxidative Stress, UV Radiation, and Temperature

The increased UV radiation and oxygen solubility occurring in polar regions favors the formation of ROS, which may cause damage to cellular macromolecules, i.e., DNA, RNA, proteins, and lipids (Cabiscol et al., 2000; Casanueva et al., 2010). The damaging effects of ROS are counteracted by antioxidant defenses. Several genes encoding predicted enzymes responsible for diminishing toxic ROS levels (glutaredoxin-related proteins, catalases, and lipoate synthase) were found in four of the *Polaromonas* spp. plasmids (pE3SP1, pE5SP1, pE10SP1, and pH6NP1).

Three of the analyzed plasmids (pE3SP1, pE5SP1, pE10SP1) carry four genes (two in pE3SP1) encoding putative glutaredoxins (COG0278) homologous to that of *Marinobacter psychrophilus* 20041 [GenBank: AKO52554] (Supplementary Table S9). It was previously shown that related enzymes repair oxidative damage in proteins containing cysteine residues by reducing the oxidized thiol groups (Ezraty et al., 2017). Moreover, plasmid pW11NP2 encodes a putative glutathione *S*-transferase (COG0625) (Supplementary Table S9) similar to the enzyme encoded by pAZKH of *Azoarcus* sp. KH32C [GenBank: BAL27334]. Glutathione *S*-transferases participate in the metabolism of glutathione – a low molecular weight thiol compound involved in glutaredoxin recycling and glutathionylation – which may occur under conditions of oxidative stress. It is hypothesized that glutathionylation prevents overoxidation of proteins and enables their reduction back to the native state (Masip et al., 2006).

Within pH6NP1, there are two genes encoding ubiquitous ROS-removing enzymes – catalase-peroxidase (COG0376) and catalase (COG0753) (Supplementary Table S9), homologous to catalase/hydroperoxidase HPI (I) encoded by a megaplasmid of *Methylobacterium extorquens* AM1 [GenBank: ACS43813] and a catalase family protein of *Hydrogenophaga* sp. RAC07 [GenBank: AOF88195], respectively. These enzymes act to minimize oxidative damage caused by toxic hydrogen peroxide by converting it into water and oxygen (Cabiscol et al., 2000).

Another ROS defense system utilizes lipoic acid, a common coenzyme with redox activity that acts as an antioxidant and free-radical scavenger. Lipoic acid synthesis is carried out by two enzymes – octanoyl transferase (LipB) and a lipoate synthase (LipA) (COG0320) (Spalding and Prigge, 2010). Proteins related to LipA are encoded by plasmids pE3SP1, pE5SP1, and pE10SP1 (Supplementary Table S9), and they show the highest sequence similarity to lipoate synthase of *Collimonas arenae* Cal35 [GenBank: AIY43929]. None of the aforementioned plasmids carry genes for LipB homologs, although this protein could be encoded by the chromosome of the host strains.

**FIGURE 4:**
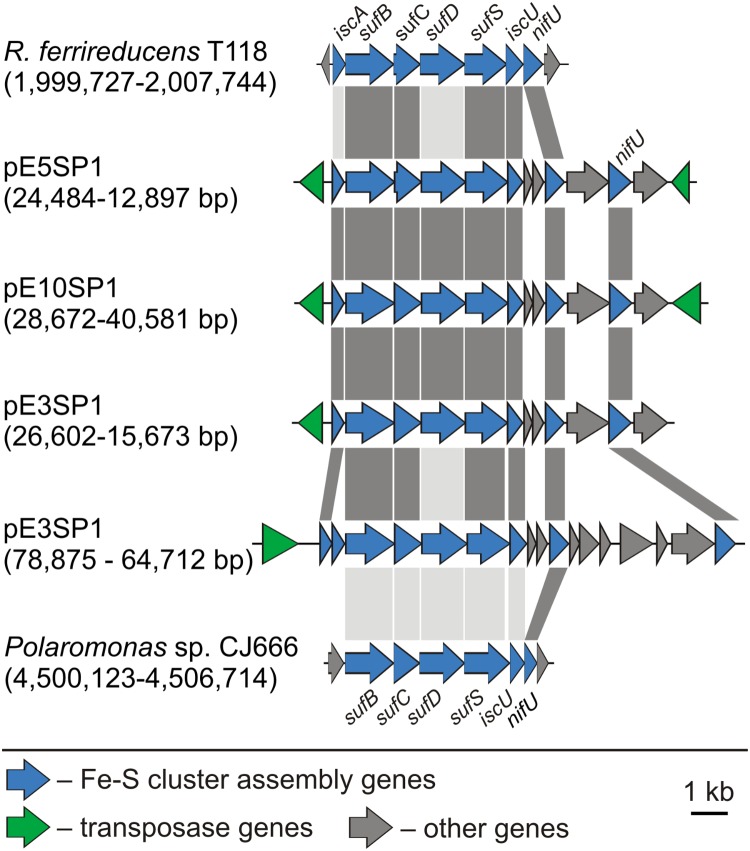
Comparative analysis of genetic modules involved in [Fe-S] cluster assembly. The coordinates of the gene clusters identified in *Polaromonas* plasmids, as well as the chromosomes of *Rhodoferax ferrireducens* T118 (GenBank: CP000267) and *Polaromonas* sp. CJ666 (GenBank: CP000316) are shown in parentheses. The gray-shaded areas connect genes encoding proteins sharing ≥70% (dark gray) or 40–70% (light gray) aa identity, respectively.

Several *Polaromonas* spp. plasmids also carry gene clusters encoding proteins putatively involved in the molecular assembly of iron–sulfur [Fe-S] clusters, important cofactors in many proteins. Four groups of *isc*-, *suf*-, and *nif*-like genes (in various combinations) were identified in plasmids pE3SP1, pE5SP1, and pE10SP1 (Supplementary Table S9). Similar *loci* are present in the chromosomes of *Polaromonas* sp. JC666 and *R. ferrireducens* T118 [GenBank: CP000316 and CP000267, respectively] (**Figure 4**). The first gene in each *locus* encodes a truncated IscA domain-containing protein (COG0316) most similar to [Fe-S] cluster assembly accessory protein [GenBank: WP_096698310] or [Fe-S] cluster insertion protein ErpA [GenBank: WP_096698577] of *Polaromonas* sp. AER18D-145. In their C-terminal regions, the IscA-like proteins contain characteristic motif with three conserved Cys residues (CX_42_DX_20-21_CGC) (Ayala-Castro et al., 2008). The biological role of these proteins is unclear, although it is hypothesized that they are components of the [Fe-S] cluster carriers (Py and Barras, 2010).

Downstream of the putative *iscA* genes (plasmids pE3SP1, pE5SP1, and pE10SP1), genes encoding SufB, SufC, SufD, and SufS homologs (COG0719, COG0396, COG0719, and COG0520, respectively) are present (Supplementary Table S9), showing homology to the respective proteins Bpro_4269-4272 of *Polaromonas* sp. JS666 [GenBank: ABE46161-4]. The SufB, SufC, and SufD proteins form a complex, which is assumed to constitute an assembly center for the creation of [Fe-S] clusters and to stimulate SufS and SufE activity (Py and Barras, 2010). SufS is a cysteine desulfurase, which, together with SufE, forms a sulfur transfer system (Ayala-Castro et al., 2008). The plasmid-encoded SufS homologs contain a conserved domain and a C-terminal sequence motif RXGHHC, characteristic of group II SufS enzymes (Mihara et al., 1997; Ayala-Castro et al., 2008). These gene clusters also encode two U-type scaffold proteins that may serve as platforms for [Fe-S] cluster assembly: (i) IscU (COG0822), with homology to an [Fe-S] assembly protein [GenBank: ABE46165] of *Polaromonas* sp. JS666 and (ii) NifU (COG0694), which is similar to NifU family proteins [GenBank: WP_096698302 and WP_096698299, respectively] of *Polaromonas* sp. AER18D-145 (Supplementary Table S9).

Other enzymes involved in the assembly of [Fe-S] clusters are glutaredoxins. Their proposed role is in the storage or transfer of [Fe-S] clusters, sensing their cellular level, and facilitating cluster assembly by the reduction of disulfides on scaffold proteins (Suf, Nif, and Isc) or acceptor proteins (Bandyopadhyay et al., 2008). Glutaredoxin-encoding genes were identified among the aforementioned antioxidant genes present in the *Polaromonas* plasmids (Supplementary Table S9).

In addition to ROS, UV radiation may cause breakage of the DNA backbone. In response to such events, DNA polymerase V (PolV) catalyzes error-prone DNA synthesis that can bypass DNA lesions and help maintain genome integrity (Pham et al., 2001). Interestingly, plasmid pH6NP1 carries a gene encoding UmuC (COG0389), a component of PolV (Supplementary Table S9). The *umuDC* genes are common in plasmids of Arctic and Antarctic bacteria, which indicate their importance in adaptation to polar environments (Dziewit and Bartosik, 2014).

One of the most significant life-limiting factors in polar regions is the permanently low (usually subzero) temperature (D’Amico et al., 2006). Under these conditions, mechanisms to maintain the fluidity of cell membranes are essential (Casanueva et al., 2010). In psychrophilic bacteria, specific enzymes involved in the metabolism of lipids and fatty acids help protect the integrity of cell membranes (Chattopadhyay, 2006). In one of the analyzed plasmids, pW11NP2, a gene encoding a predicted long-chain-fatty-acid-CoA ligase (COG0318) was identified (Supplementary Table S9). We speculate that this ligase along with other chromosomally encoded enzymes may be involved in remodeling membrane lipids to permit growth in freezing temperatures.

#### Transport and Metabolism of Organic Compounds

Nutrient deficiency is another serious problem facing bacteria inhabiting cold environments. Therefore, these microorganisms require (i) efficient transport systems for the acquisition of available compounds and (ii) enzymes to metabolize a wide range of substrates. This makes polar bacteria nutrient opportunists rather than obligatory specialists (Simon et al., 2009; De Maayer et al., 2014; Dsouza et al., 2015). Plasmids carrying the genes necessary for the sequestration and metabolism of different compounds may help bacteria to colonize nutrient-limiting ecological niches.

Within the analyzed *Polaromonas* plasmids, genes encoding three putative nutrient transport systems were identified (Supplementary Table S9): (i) the periplasmic component of an ABC-type branched-chain amino acid transport system (COG0683) of pW11NP2 (similar to an ABC transporter substrate-binding protein of *Thauera* sp. MZ1T [GenBank: ACK54136]), (ii) an ABC system composed of a periplasmic protein (COG0834), permease (COG0765), and ATPase (COG1126) of pE10SP1 (related genes, conserved in sequence and synteny, are found in *Rhodoferax saidenbachensis* DSM 22694 [GenBank: APW43001, APW43002, and APW43003, respectively]), and (iii) four components of polyamine transport system Pot (COG3842, COG0687, COG1176, and COG1177) of pE10SP1 (similar to proteins encoded by a gene cluster of *Polaromonas* sp. JS666 [GenBank: ABE42965–ABE42965]).

Several *Polaromonas* spp. plasmids also encode enzymes potentially involved in the metabolism of amino acids and carbohydrates (Supplementary Table S9). These include enzymes involved in the conversion of amino acids: (i) small IlvH (COG0440) and large IlvB (COG0028) subunits of acetolactate synthase of pE3SP1 (similar to those of *Fibrobacter succinogenes* subsp. *succinogenes* S85 [GenBank: ACX76297 and ACX76298, respectively]), an enzyme catalyzing the first step in branched-chain amino acid synthesis (Pue and Guddat, 2014), (ii) selenocysteine lyase (COG0520) of pE10SP1 (similar to a cysteine desulfurase-like protein of *R. saidenbachensis* DSM 22694 [GenBank: APW43000]), and (iii) Asp/Glu/hydantoin racemase (COG4126) in pE10SP1 (similar to Asp/Glu racemase of *P. naphthalenivorans* CJ2 [GenBank: ABM38571]) (Supplementary Table S9). The predicted proteins involved in carbohydrate metabolism include (i) glucose/arabinose dehydrogenases (COG2133) of pE3SP1, pE5SP1, pE10SP1, pE19SP1, and pW11NP2 (similar to L-sorbosone dehydrogenase of *P. naphthalenivorans* CJ2 [GenBank: ABM35718]) and (ii) a putative polysaccharide deacetylase of pE10SP1 (homologous to a polysaccharide deacetylase family protein of *Hydrogenophaga* sp. RAC07 [GenBank: AOF83940]) (Supplementary Table S9).

In addition, two of the plasmids (pE3SP1 and pE10SP1) encode aconitate hydratase, an enzyme of the TCA cycle that converts citrate into isocitrate (both proteins are homologous to aconitate hydratase of *M. psychrophilus* 20041 [GenBank: AKO52539]). Plasmid pH6NP1 carries a gene encoding a putative acetyl esterase/lipase (COG0657) (similar to lipase LipP of Alaskan psychrophilic strain *Pseudomonas* sp. B11-1 [GenBank: AF034088]) (Choo et al., 1998) (Supplementary Table S9).

Other identified genes with adaptive potential encode (i) periplasmic DMSO/TMAO reductase (COG2041) of plasmid pE3SP1 (similar to sulfite oxidase of *Rhizobacter gummiphilus* NS21 [GenBank: ARN19249]), (ii) rhodanese-related sulfurtransferase (COG0607) of pE3SP1, involved in sulfane sulfur transfer between organic compounds (similar to sulfurtransferase of *M. psychrophilus* 20041 [GenBank: AKO54292]), (iii) GTP cyclohydrolase I (COG0302) of pE3SP1, catalyzing the first step in folic acid biosynthesis in bacteria (similar to GTP cyclohydrolase I of *Janthinobacterium* sp. LM6 [GenBank: AQR66946]), (iv) cyanuric acid hydrolase of pE10SP1, catalyzing a hydrolytic ring amide bond cleavage reaction (similar to cyanuric acid amidohydrolase of *Alcanivorax xenomutans* P40 [GenBank: ARB44603]), (v) two enzymes involved in the metabolism of nucleotides, i.e., dihydroorotase (or related cyclic amidohydrolase) (COG0044) and ureidoglycolate lyase (COG3194) of pE10SP1 (similar to dihydropyrimidinase and ureidoglycolate hydrolase of *R. saidenbachensis* DSM 22694 [GenBank: APW42999 and APW44816, respectively]), and (vi) pimeloyl-ACP methyl ester carboxylesterase (COG0596) of pH6NP1 (similar to an alpha/beta hydrolase family protein of *Hydrogenophaga* sp. RAC07 [GenBank: AOF86140]) (Supplementary Table S9).

#### Transport of Metal Ions and Resistance to Heavy Metals

Bacteria living on glaciers may be exposed to toxic metals accumulated on the glacial surface (Hur et al., 2007; Singh et al., 2013; Lokas et al., 2016). Five *Polaromonas* spp. plasmids (pE3SP1, pE5SP1, pE10SP1, pE19SP1, and pH6NP1) carry genes encoding transporters of metal ions (e.g., cadmium, manganese, iron, zinc). The presence of these transporters may increase metal uptake and/or enable metal efflux from the cell, both of which could be beneficial to the bacterium.

The highest number of genetic modules linked with metal transport and resistance was found within pH6NP1. This plasmid carries (i) a predicted mercury resistance genetic module encoding a putative MerR transcriptional regulator, Hg^2+^ transporters MerT, MerP, and MerC, and mercuric reductase MerA, which converts toxic Hg^2+^ ions into Hg^0^; (ii) a gene encoding a predicted MntH transporter of the NRAMP family (COG1914), similar to the iron transporter of *Sulfuriferula* sp. AH1 [GenBank: ARU31281] – expressed in low Mn^2+^ concentrations and responsible for the uptake of Mn^2+^ ions (Que and Helmann, 2000); and (iii) a gene encoding an efflux pump of the cation diffusion facilitator (CDF) family (COG0053) with homology to a cation transporter of *Variovorax* sp. PAMC 28711 [GenBank: AMM26068] (Supplementary Table S9). CDF family transporters are ubiquitous in bacteria, and they play an important role in homeostasis and resistance to several divalent metal ions, i.e., Cd^2+^, Co^2+^, Cu^2+^, Fe^2+^, Mn^2+^, Ni^2+^, and Zn^2+^. For example, CzrB of *Staphylococcus aureus* 912 confers tolerance to Zn^2+^ and Co^2+^, while DmeF of *Wautersia metallidurans* CH34 mediates resistance against Co^2+^ (Kuroda et al., 1999; Munkelt et al., 2004).

Plasmids pE5SP1, pE10SP1, and pE19SP1 carry genes encoding proteins with a ZnuA domain (COG0803) (Supplementary Table S9) (related proteins are components of a zinc-uptake *znuABC* system controlling cytoplasmic Zn^2+^ levels), with sequence similarity to a periplasmic solute binding protein of *P. naphthalenivorans* CJ2 [GenBank: ABM36458]. Moreover, these plasmids also carry genes encoding proteins homologous to TonB-dependent outer membrane receptors (COG1629) (Supplementary Table S9), similar to a related protein of *Polaromonas* sp. JS666 [GenBank: ABE43619]. Proteins of this type are very common in bacteria and are responsible for binding and import of a wide range of compounds, including inorganic ions, siderophores, vitamin B_12_, and carbohydrates (Noinaj et al., 2010).

Heavy metal resistance genes were also found within plasmids pE3SP1 and pE10SP1 (Supplementary Table S9). Both encode proteins homologous to the heavy metal translocating P-type ATPase ZntA (COG2217), encoded by plasmid pPNAP02 of *P. naphthalenivorans* CJ2 [GenBank: ABM39819]. ZntA transporters confer resistance to various divalent ions, including Cd^2+^, Co^2+^, Cu^2+^, Ni^2+^, Pb^2+^, and Zn^2+^ (Hou et al., 2001; Hou and Mitra, 2003).

### Functional Analysis of Heavy Metal Ion Resistance Modules

We tested whether the predicted genes and gene clusters carried by the *Polaromonas* plasmids are able to confer resistance to heavy metals. Initially, the effect of Cd^2+^, Co^2+^, Cu^2+^, Hg^2+^, Mn^2+^, Ni^2+^, and Zn^2+^ ions on *Polaromonas* strains hosting these plasmids was examined. These metals were selected based on the predicted metal-specificity of the putative resistance genes identified within the plasmids. MIC values were determined to establish the level of metal resistance of the plasmid-bearing strains (**Table 2**).

**Table 2:** MIC values [mM] for heavy metal ions of plasmid-harboring *Polaromonas* strains.

Origin of the strain	*Polaromonas* strain	Metal ion					
		
		Cd^2+^	Co^2+^	Cu^2+^	Hg^2+^	Mn^2+^	Ni^2+^	Zn^2+^
Antarctic	E3S	<1.0	<1.0	**3.0**	<0.05	<1.0	<1.0	<1.0
	E5S	<1.0	<1.0	<1.0	<0.05	3.0	<1.0	<1.0
	E10S	<1.0	<1.0	<1.0	<0.05	2.0	**2.0**	<1.0
	E19S	<1.0	<1.0	<1.0	<0.05	<1.0	<1.0	<1.0
Arctic	H1N	<1.0	**2.0**	**2.0**	<0.05	7.0	<1.0	**4.0**
	H6N	<1.0	**2.0**	**4.0**	<0.05	8.0	**2.0**	**4.0**
	H8N	<1.0	**2.0**	**2.0**	<0.05	9.0	**2.0**	**3.0**
	W5N	<1.0	**2.0**	**2.0**	<0.05	7.0	**2.0**	**2.0**
	W9N	<1.0	<1.0	<1.0	<0.05	2.0	<1.0	**2.0**
	W10N	<1.0	**2.0**	**2.0**	<0.05	5.0	<1.0	**3.0**
	W11N	<1.0	**2.0**	<1.0	<0.05	2.0	<1.0	**3.0**


*Resistance phenotypes (designated on the basis of MIC values) are shown in bold.*

None of the strains (including strain H6N, which carries a predicted *mer* module) exhibited resistance to mercury ions [the Hg^2+^ concentration cut-off was 0.05 mM – twofold lower than that proposed by Nieto et al. (1987)] (**Table 2**). In the case of the strains E5S, E10S, and E19S, the ZnuA plasmid-encoded proteins did not confer a zinc-tolerant phenotype. Therefore, no correlation between the presence of *mer* and *znuA*-like genes and the resistance phenotypes of their hosts was observed. Moderate levels of resistance to Co^2+^, Cu^2+^, Ni^2+^, and Zn^2+^ ions were observed for *Polaromonas* sp. H6N (**Table 2**). These phenotypes may result from the presence of the *pH6NP1_p047* gene, encoding a putative efflux pump of the CDF family, on the plasmid pH6NP1, which resides in this strain. The highest level of Cu^2+^ tolerance was observed for *Polaromonas* sp. E3S (**Table 2**). This phenotype may result from the presence of the *pE3SP1_p089* gene, encoding a ZntA family protein, within plasmid pE3SP1. Interestingly, another analyzed strain (E10S), also carrying a plasmid encoding a ZntA protein, showed low-level resistance to Ni^2+^, but not to Cu^2+^ ions (**Table 2**).

**FIGURE 5:**
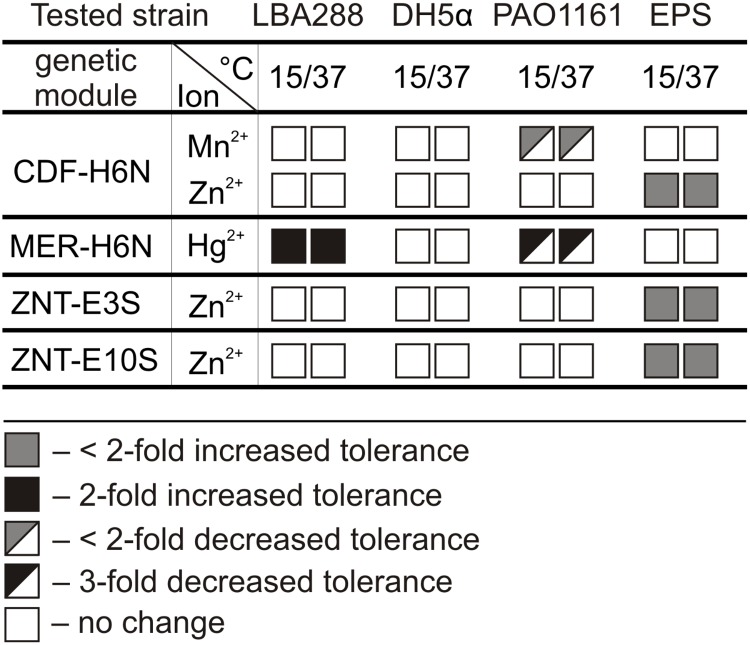
The influence of predicted CDF, MER, and ZNT resistance modules on the heavy metal tolerance of *Agrobacterium tumefaciens* LBA288, *Escherichia coli* DH5α, *Pseudomonas aeruginosa* PAO1161, and *V. paradoxus* EPS. The resistance phenotypes of the transformed strains were tested at both 15 and 37°C. CDF-H6N – cadmium, cobalt, copper, manganese, nickel, and/or zinc resistance module of plasmid pH6NP1; MER-H6N – mercury resistance module of plasmid pH6NP1; ZNT-E3S and ZNT-E10S – P-type heavy metal-transporting ATPase of plasmids pE3SP1 and pE10SP, respectively.

Since mobile plasmids can be maintained in different bacteria, the activity of the predicted resistance genes was tested in other host strains. The following genes/modules were amplified by PCR and cloned within the broad host range mobilizable vector pBBR1 MCS-2: (i) the ZnuA component of the *znuABC* system of pE5SP1 and pE19SP1 – ZNU modules, (ii) the P-type heavy metal-transporting ATPase ZntA of pE3SP1 and pE10SP1 – ZNT modules, (iii) the CDF family divalent metal cation transporter of pH6NP1 – CDF module, and (iv) the mercury resistance module of pH6NP1 *–* MER module (Supplementary Table S2). Each of the pBBR1 MCS-2 derivatives was introduced into *A. tumefaciens* LBA288 (*Alphaproteobacteria*), *V. paradoxus* EPS (*Betaproteobacteria*), *E. coli* DH5α (*Gammaproteobacteria*), and *P. aeruginosa* PAO1161 (*Gammaproteobacteria*). The resistance phenotypes of the transformed strains were then examined. MIC assays were conducted at both the optimal growth temperature for each strain (30 or 37°C) and at 15°C, which is the suitable growth temperature for *Polaromonas* spp. – in case gene expression was temperature dependent (**Figure 5** and Supplementary Table S10).

The MER module of pH6NP1 was found to be active in *A. tumefaciens* LBA288 and *P. aeruginosa* PAO1161. Interestingly, the introduction of this module into *A. tumefaciens* resulted in a twofold increase in the MIC for Hg^2+^ ions, while the same module caused a threefold increase in sensitivity to these ions in *P. aeruginosa*. Introduction of the CDF module and both ZNT modules into *V. paradoxus* EPS caused a slight increase in tolerance to Zn^2+^ ions. In addition, the presence of the CDF module in *P. aeruginosa* PAO1161 resulted in slightly decreased tolerance to Mn^2+^ ions (**Figure 5** and Supplementary Table S10). Neither of the ZNU modules was active in the tested hosts (Supplementary Table S10). This analysis also revealed that the activity of these resistance determinants is not temperature dependent.

## Discussion

This study has provided the first insight into the plasmidome of psychrotolerant bacteria of the genus *Polaromonas*, which are very common in the Arctic and Antarctic regions (Anesio et al., 2017). It resulted in the genomic and functional characterization of 13 novel plasmids, which is a significant contribution to our knowledge of extrachromosomal replicons of psychrophilic and psychrotolerant bacteria (Dziewit and Bartosik, 2014).

According to the origin of their REP regions, 12 of the analyzed plasmids were classified into 5 families – RepA_C, Rep3, RepL, RPA, and TrfA. Comparative analysis revealed that two RepA_C-type plasmids (pH8NP1 and pW9NP1) are the first replicons carrying modules of this REP family to be identified in cold-active bacteria. Another interesting case is the REP module of plasmid pW11NP1, which is not only unique among cold-active bacteria but also represents a novel plasmid replication system.

Our analysis revealed that all of the studied *Polaromonas* spp. plasmids are narrow host range replicons, only able to replicate in bacteria of the genera *Polaromonas* and *Variovorax* (*Comamonadaceae* family) – both of which are ubiquitous in polar regions (Ciok et al., 2016). This suggests that plasmid transfer involving bacteria of the *Comamonadaceae* family in polar regions may be limited by narrow host range of their replicons. Interestingly, the majority of plasmids of Arctic and Antarctic bacteria examined in our previous studies also had a narrow host range (Dziewit et al., 2013a,b; Ciok et al., 2016; Romaniuk et al., 2018), which may suggest that this phenomenon is more common.

Besides REPs, the conserved backbones of the *Polaromonas* plasmids also comprised PAR, TA, and MOB modules. The most numerous were TA systems (20 *loci* in 7 plasmids). Interestingly, multiple TA systems (4–5) were present within three large (between 65.5 and 101 kb) plasmids (pE3SP1, pE5SP1, and pE10SP1) of Antarctic strains, while no such systems were identified in another large plasmid pH6NP1 (82.5 kb) originating from an Arctic strain. As previously mentioned, TA systems encode a toxin and antitoxin. Antitoxins are less stable components, so their concentration is decreased in TA(plasmid)-less cells. In consequence, the liberated toxins act on their cellular targets resulting in a bacteriostatic or bactericidal effect (Leplae et al., 2011; Unterholzner et al., 2013). Therefore, TA systems may be considered selfish DNA modules, which ensure their persistence in a bacterial population by the elimination of TA(plasmid)-less cells (Van Melderen and Saavedra De Bast, 2009).

Since many bacterial plasmids carry adaptive genes that ensure the predominance of their hosts in specific ecological niches, the loss of these replicons (or their elements) may be disadvantageous for the bacteria (diCenzo et al., 2016; Czarnecki et al., 2017; diCenzo and Finan, 2017). Our analysis revealed that the large Antarctic plasmids pE3SP1, pE5SP1, and pE10SP1 are especially rich in phenotypic modules that are presumably beneficial to their host strains. Interestingly, all of these plasmids carry gene clusters encoding proteins putatively involved in molecular assembly of iron–sulfur [Fe-S] clusters, which are important cofactors in a number of proteins. It is noteworthy that these plasmids also encode a large number of transposases, i.e., enzymes with great recombination potential. Therefore, the aforementioned TA systems may prevent the loss of adaptive genes and act to protect plasmid genome integrity.

To our knowledge, the plasmids pE3SP1, pE5SP1, and pE10SP1 are the first reported examples of extrachromosomal replicons carrying genes encoding proteins involved in [Fe-S] cluster assembly. These genes may represent an important adaptation to polar environments. In these regions, the UV radiation and oxygen solubility are increased, which favors the formation of ROS that can damage cellular macromolecules (Cabiscol et al., 2000; Casanueva et al., 2010). Proteins containing [Fe-S] clusters are especially vulnerable to ROS, so are highly unstable in the presence of oxygen (Kiley and Beinert, 2003; Imlay, 2006; Outten, 2007).

It is estimated that the correct functioning of up to 100 bacterial proteins depends on [Fe-S] clusters, and many of these are crucial for cell survival. Proteins containing [Fe-S] clusters take part in electron transfer, sensing environmental conditions, and controlling the structure of other proteins (Frazzon and Dean, 2003). To date, three [Fe-S] cluster assembly pathways have been identified in bacteria: (i) Isc (iron sulfur cluster), (ii) Suf (sulfur formation cluster), and (iii) Nif (nitrogen fixation cluster) (Ayala-Castro et al., 2008). Each system is thought to play a different, although potentially overlapping, role in cell physiology: the Isc system is used for [Fe-S] cluster assembly in housekeeping proteins, the Suf system is active under stress conditions, while the Nif system is required for the assembly of [Fe-S] clusters in specific enzymes. It was also shown that the expression of genes of the Isc and Suf systems is induced in response to ROS (Ayala-Castro et al., 2008). As [Fe-S] cluster assembly is an essential cellular function, we hypothesize that plasmid-encoded proteins involved in this process may cooperate with these chromosomally encoded systems.

Interestingly, the *Polaromonas* spp. plasmids lack one component of the [Fe-S] cluster assembly – the *sufE* gene. Since [Fe-S] cluster assembly is a basic cellular process, we speculate that *sufE* genes (and possibly other genes encoding proteins involved in [Fe-S] cluster assembly) may be present within the hosts’ chromosomes. In this situation, abovementioned genetic modules carried on plasmids constitute an additional (to chromosomal ones) copies of genes, which may increase the cellular pool of proteins involved in [Fe-S] cluster assembly, and this could be beneficial to the host under specific conditions.

Low temperature, nutrient limitation, and the possible presence of toxic compounds and/or heavy metals are important factors that can limit life in polar regions (Martianov and Rakusa-Suszczewski, 1990; D’Amico et al., 2006; Kejna et al., 2013; Kosek et al., 2017). Our analysis revealed that *Polaromonas* spp. plasmids can assist the adaptation of their hosts to these adverse conditions. An abundant group of genes carried by *Polaromonas* spp. plasmids encode enzymes involved in cellular metabolism and transporters of various substrates (including organic and inorganic compounds). The acquisition of a plasmid carrying such genes may enable the uptake and usage of a broader range of compounds as metabolic substrates in nutrient-poor environments.

The analyzed plasmids also contain putative heavy metal resistance genes, which may be considered a response to the raised levels of these elements in polar regions. Contamination of Arctic and Antarctic regions by different pollutants, such as heavy metals, is an emerging problem. A natural source of heavy metals in these regions is volcanic eruptions, e.g., those occurring on Iceland. Pollutants are also carried to polar regions by airflows from industrial areas in mid- and low-latitudes (Bard, 1999). We identified six predicted heavy metal resistance modules in the analyzed plasmids and tested their functionality in heterologous hosts. The activity of four, i.e., MER and CDF of pH6NP1, and ZNT of pE3SP1 and pE10SP1, was experimentally proven.

One interesting case was the MER module of pH6NP1, which conferred mercury (Hg^2+^) resistance to cells of *A. tumefaciens* LBA288 but increased the sensitivity of *P. aeruginosa* PAO1161 to these ions (**Figure 5** and Supplementary Table S10). The *mer* operon contains the *merT*, *merP*, and *merC* genes, which encode transporters that enable the uptake of toxic Hg^2+^ ions into the cell, and the gene *merA* encoding mercuric reductase, an enzyme responsible for volatilizing mercury as Hg^0^. We speculate that the observed phenotype in the PAO1161 strain is due to the weak (or lack of) expression of the *merA* gene. Thus the insufficient reduction of Hg^2+^ into the less toxic Hg^0^ by MerA and further accumulation of mercury ions within the cell may result in a toxic effect. A similar phenotype was previously observed for Tn*5563*a, which carries a partial MER module lacking the *merA* gene (Dziewit et al., 2015).

Mercury resistance modules are ubiquitous in bacterial genomes and are frequently localized within mobile genetic elements (e.g., plasmids and transposons), which favors their further dissemination (Barkay et al., 2003). Interestingly, an analysis of the distribution of mercury resistance genes among cold-active bacteria originating from Arctic and Antarctica, i.e., regions with limited anthropopression, revealed that *merA* genes are very common, even if the mercury concentration in the sampled environment is negligible (Møller et al., 2014).

Functional analysis of another resistance module, CDF of plasmid pH6NP1, revealed that it caused a slight increase in tolerance to Zn^2+^ in *V. paradoxus* EPS cells, whereas it made *P. aeruginosa* PAO1161 more sensitive to Mn^2+^. The CDF modules encode a divalent cation efflux pump, involved in metal homeostasis and resistance (Cubillas et al., 2013). Our observations are in agreement with previous findings which suggest that the introduction of an exogenous heavy metal resistance module into bacterial cells (especially genes encoding transmembrane pumps) does not always produce increased resistance. Indeed, it may often adversely affect the metal homeostasis of the cell (e.g., by altering ion fluxes and their intracellular concentration), resulting in increased sensitivity to particular heavy metal ions (Dziewit et al., 2015; Romaniuk et al., 2018).

In this study, nucleotide sequences of chromosomes of the analyzed *Polaromonas* strains were not determined. Therefore, to evaluate a possible adaptive value of polar *Polaromonas* spp. plasmids, a BLASTP search for 89 plasmid-encoded proteins (listed in Supplementary Table S9), considered as having adaptive potential, versus chromosome-encoded proteins of *Polaromonas* sp. JS666 and *P. naphtalenivorans* CJ2, was carried out. For 11 proteins, no homologs were found within the screened chromosomes. These were: (i) pW11NP2_p035 (glutathione *S*-transferase), (ii) pE10SP1_p064 (cyanuric acid hydrolase), (iii) pH6NP1_p054 (MntH transporter), and (iv–xi) pE3SP1_p017, pE3SP1_p070, pE5SP1_p017, pE10SP1_p040, pE3SP1_p015, pE3SP1_p063, pE5SP1_p015, and pE10SP1_p042 ([Fe-S] cluster biogenesis scaffold protein Nfu/NifU). The fact that genes encoding these proteins were found exclusively within the analyzed plasmids suggests that carrying such plasmids may contribute to better environmental adaptation. Homologs of the remaining 78 proteins are encoded within the screened *Polaromonas* chromosomes, which may suggest that the corresponding genes are present also within the chromosomes of the analyzed cold-active strains. In that case, the presence of additional gene copies within extrachromosomal replicons could result in higher cellular dosages of the particular proteins, which may strengthen the overall cell adaptation to environmental conditions.

**FIGURE 6:**
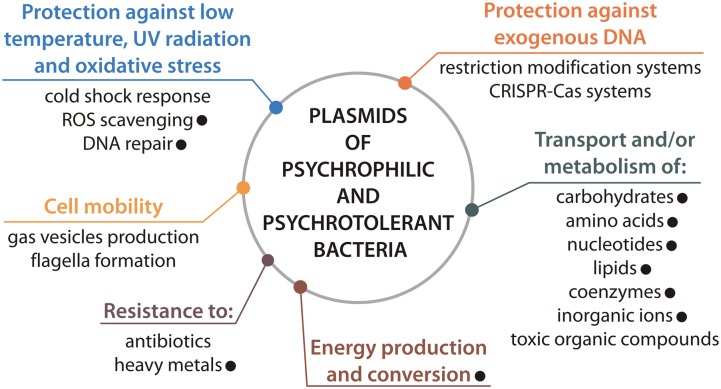
Plasmid-encoded features contributing to adaptation of cold-active bacteria to polar environments. The presence of particular feature within *Polaromonas* plasmids is indicated by black dot.

In summary, the findings of this study show that plasmids may play an important role in the adaptation of *Polaromonas* spp. to extreme Arctic and Antarctic conditions (**Figure 6**). Many of the characterized *Polaromonas* plasmids carry beneficial genes that increase the overall tolerance of their hosts to harsh environmental conditions, including oxidative stress, the presence of toxic elements, and nutrient limitation. This is in line with our previous meta-analysis of plasmids of psychrophiles and psychrotolerants (Dziewit and Bartosik, 2014), and all together reflects the importance of plasmids in adaptation of bacteria to cold environments (**Figure 6**).

## Author Contributions

LD and AC conceived and designed the experiments, analyzed the data, and wrote the manuscript. JGr and MZ isolated strains, performed the field work, and prepared descriptions of the sampling sites and sampling methodology. JGa, RG, JGr, and LD sequenced the plasmids. AC and KB performed the functional analyses. AC, KB, and PD annotated the plasmid sequences. AC performed all other bioinformatic analyses. LD, MZ, and DB contributed reagents, materials, analysis tools. DB revised and modified the manuscript to its final version. All authors read and approved the submitted version.

## Conflict of Interest Statement

The authors declare that the research was conducted in the absence of any commercial or financial relationships that could be construed as a potential conflict of interest.

## Footnotes

1 http://blast.ncbi.nlm.nih.gov/Blast.cgi
