# Supplementary material for: Plasmids of Psychrotolerant Polaromonas spp. Isolated From Arctic and Antarctic Glaciers – Diversity and Role in Adaptation to Polar Environments
Source: Front Microbiol. 2018 Jun 18;9:1285. doi: 10.3389/fmicb.2018.01285 (PMC6015842; doi:10.3389/fmicb.2018.01285)
Supplement: Supplementary file 1 [file Table_1.PDF]

Supplementary Material

Plasmids of Psychrotolerant *Polaromonas* spp. Isolated from Arctic and Antarctic Glaciers – Diversity and Role in Adaptation to Polar Environments

Anna Ciok[1], Karol Budzik[1], Marek K. Zdanowski[2], Jan Gawor[3], Jakub Grzesiak[2], Przemyslaw Decewicz[1], Robert Gromadka[3], Dariusz Bartosik[1], Lukasz Dziewit[1*]

*** Correspondence:** Dr. Lukasz Dziewit: ldziewit@biol.uw.edu.pl

TABLE S1. Summary of genomes of *Polaromonas* spp.

Strain	Level (number of replicons or scaffolds or contigs)	GenBank accession number	Estimated genome size (Mb)*	Estimated GC content (%)*	Reference
P. glacialis R3-9	scaffold (27)	JMDZ01000001-JMDZ01000059	5.22	62.30	Wang et al., 2014
P. jejuensis NBRC 106434	contig (79)	BCYO01000001-BCYO01000079	5.13	62.90	None
P. naphthalenivorans CJ2	complete genome (9)	CP000529 (chromosome)<br>CP000530 (pPNAP01)<br>CP000531 (pPNAP02)<br>CP000532 (pPNAP03)<br>CP000533 (pPNAP04)<br>CP000534 (pPNAP05)<br>CP000535 (pPNAP06)<br>CP000536 (pPNAP07)<br>CP000537 (pPNAP08)	5.37	61.66	Yagi et al., 2009
Polaromonas sp. 16-63-31	scaffold (57)	NCGW01000001-NCGW01000057	4.91	62.90	None
Polaromonas sp. 17-63-33	contig (53)	NCIK01000001-NCIK01000053	5.12	62.50	None
Polaromonas sp. 24-63-21	scaffold (59)	NCHP01000001-NCHP01000059	5.07	62.70	None
Polaromonas sp. 24-62-144	scaffold (518)	NCHV01000001-NCHV01000518	3.01	62.40	None
Polaromonas sp. 28-63-22	scaffold (548)	NCGI01000001-NCGI01000548	3.12	62.60	None

Polaromonas sp. 35-63-35	contig (52)	NCFQ01000001-NCFQ01000052	4.91	62.90	None
Polaromonas sp. 35-63-240	scaffold (549)	NCFW01000001-NCFW01000549	3.00	62.50	None
Polaromonas sp. 39-63-25	contig (54)	NCJH01000001-NCJH01000054	4.83	63.00	None
Polaromonas sp. 39-63-203	scaffold (568)	NCJN01000001-NCJN01000568	2.99	62.60	None
Polaromonas sp. A23	contig (51)	MUNO01000001-MUNO01000051	4.58	61.10	None
Polaromonas sp. AET17H-212	contig (86)	NBZW01000001-NBZW01000086	4.65	63.80	None
Polaromonas sp. AER18D-145	contig (135)	NBZV01000001-NBZV01000135	4.82	63.50	None
Polaromonas sp. C04	contig (45)	MUNS01000001-MUNS01000045	4.17	64.30	None
Polaromonas sp. CF318	contig (159)	AKIV01000001-AKIV01000159	5.01	64.90	Brown et al., 2012
Polaromonas sp. CG9_12	contig (7)	CCJP01000001-CCJP01000007	4.90	61.40	Smith et al., 2014
Polaromonas sp. EUR3 1.2.1	contig (3)	JIBH01000001-JIBH01000003	4.40	59.90	None
Polaromonas sp. JS666	complete genome (3)	CP000316 (chromosome)<br>CP000317 (plasmid 1)<br>CP000318 (plasmid 2)	5.90	62.00	Mattes et al., 2008
Polaromonas sp. OV174	contig (61)	FOKZ01000001-FOKZ01000061	4.53	61.30	None
Polaromonas sp. UBA4122	scaffold (172)	DFWY01000001-DFWY01000172	4.17	59.30	Parks et al., 2017
Polaromonas sp. UBA5171	scaffold (138)	DHWX01000001-DHWX01000138	4.00	59.10	Parks et al., 2017
Polaromonas sp. YR568	scaffold (22)	FPBM01000001-FPBM01000022	4.83	62.80	None

* For complete genomes the approximate value was provided.

References:

Brown, S. D., Utturkar, S. M., Klingeman, D. M., Johnson, C. M., Martin, S. L., Land, M. L., Lu Tse-Yuan S., Schadt C. W., Doktycz M. J., and Pelletier, D. A. (2012). Twenty-one genome sequences from Pseudomonas species and 19 genome sequences from diverse bacteria isolated from the rhizosphere and endosphere of *Populus deltoides*. Journal of bacteriology, 194(21), 5991-5993.

Mattes, T. E., Alexander, A. K., Richardson, P. M., Munk, A. C., Han, C. S., Stothard, P., and Coleman, N. V. (2008). The genome of *Polaromonas* sp. strain JS666: insights into the evolution of a hydrocarbon-and xenobiotic-degrading bacterium, and features of relevance to biotechnology. Applied and environmental microbiology, 74(20), 6405-6416.

Parks, D. H., Rinke, C., Chuvochina, M., Chaumeil, P. A., Woodcroft, B. J., Evans, P. N., Hugenholtz P., and Tyson, G. W. (2017). Recovery of nearly 8,000 metagenome-assembled genomes substantially expands the tree of life. Nature microbiology, 2(11), 1533.

Smith, H. J., Foreman, C. M., and Ramaraj, T. (2014). Draft genome sequence of a metabolically diverse Antarctic supraglacial stream organism, *Polaromonas* sp. strain CG9_12, determined using Pacific Biosciences single-molecule real-time sequencing technology. Genome announcements, 2(6), e01242-14.

Wang, Z., Xulu Chang, X. Y., Pan, L., and Dai, J. (2014). Draft genome sequence of *Polaromonas glacialis* strain R3-9, a psychrotolerant bacterium isolated from Arctic glacial foreland. Genome announcements, 2(4).

Yagi, J. M., Sims, D., Brettin, T., Bruce, D., and Madsen, E. L. (2009). The genome of *Polaromonas naphthalenivorans* strain CJ2, isolated from coal tar-contaminated sediment, reveals physiological and metabolic versatility and evolution through extensive horizontal gene transfer. Environmental microbiology, 11(9), 2253-2270.
