# Supplementary material for: Plasmids of Psychrotolerant Polaromonas spp. Isolated From Arctic and Antarctic Glaciers – Diversity and Role in Adaptation to Polar Environments
Source: Front Microbiol. 2018 Jun 18;9:1285. doi: 10.3389/fmicb.2018.01285 (PMC6015842; doi:10.3389/fmicb.2018.01285)
Supplement: Supplementary file 2 [file Table_2.PDF]

# *Supplementary Material*

# Plasmids of Psychrotolerant *Polaromonas* spp. Isolated from Arctic and Antarctic Glaciers – Diversity and Role in Adaptation to Polar Environments

**Anna Ciok[1], Karol Budzik[1], Marek K. Zdanowski[2], Jan Gawor[3], Jakub Grzesiak[2], Przemyslaw Decewicz[1], Robert Gromadka[3], Dariusz Bartosik[1], Lukasz Dziewit[1*]**

*** Correspondence:** Dr. Lukasz Dziewit: ldziewit@biol.uw.edu.pl

**TABLE S2.** Plasmids used in this study.

| Plasmid name* | Characteristics | Reference or source |
|---|---|---|
| pABW1 | Km$^r$; 4.5 kb; *ori* pMB1; *oriT* RK2; *lacZα*; MCS | Bartosik et al., 1997 |
| pBBR1MCS-2 | Km$^r$; 5.1 kb; *ori* pBBR1; *oriT* RK2; *lacZα*; MCS | Kovach et al., 1994 |
| pABW1-REP-E3SP1 | Km$^r$; 6.5 kb; pABW1 derivative carrying REP module of plasmid pE3SP1 (amplified by PCR with primers LREPE3SP1 and RREPE3SP1) inserted between BamHI and EcoRI sites | This study |
| pABW1-REP-pE5SP1 | Km$^r$; 6.5 kb; pABW1 derivative carrying REP module of plasmid pE5SP1 (amplified by PCR with primers LREPE5SP1 and RREPE5SP1) inserted between BamHI and EcoRI sites | This study |
| pABW1-REP-E10SP1 | Km$^r$; 6.5 kb; pABW1 derivative carrying REP module of plasmid pE10SP1 (amplified by PCR with primers LREPE10SP1 and RREPE10SP1) inserted between BamHI and EcoRI sites | This study |
| pABW1-REP-E19SP1 | Km$^r$; 6.8 kb; pABW1 derivative carrying REP module of plasmid pE19SP1 (amplified by PCR with primers LREPE19SP1 and RREPE19SP1) inserted between BamHI and PstI sites | This study |
| pABW1-REP-H1NP1 | Km$^r$; 6.3 kb; pABW1 derivative carrying REP module of plasmid pH1NP1 (amplified by PCR with primers LREPH1NP1 and RREPH1NP1) inserted between BamHI and EcoRI sites | This study |
| pABW1-REP-H6NP1 | Km$^r$; 6.3 kb; pABW1 derivative carrying REP module of plasmid pH6NP1 (amplified by PCR with primers LREPH6NP1 and RREPH6NP1) inserted between BamHI and EcoRI sites | This study |
| pABW1-REP-H8NP1 | Km$^r$; 6.0 kb; pABW1 derivative carrying REP module of plasmid pH8NP1 (amplified by PCR with primers LREPH8NP1 and RREPH8NP1) inserted between HindIII and PstI sites | This study |
| pABW1-REP-H8NP2 | Km$^r$; 7.0 kb; pABW1 derivative carrying REP module of plasmid pH8NP2 (amplified by PCR with primers LREPH8NP2 and RREPH8NP2) inserted between BamHI and EcoRI sites | This study |
| pABW1-REP-W5NP1 | Km$^r$; 6.1 kb; pABW1 derivative carrying REP module of plasmid pW5NP1 (amplified by PCR with primers LREPW5NP1 and RREPW5NP1) inserted between BamHI and HindIII sites | This study |

| | | |
|---|---|---|
| pABW1-REP-W9NP1 | Km$^r$; 5.6 kb; pABW1 derivative carrying REP module of plasmid pW9NP1 (amplified by PCR with primers LREPW9NP1 and RREPW9NP1) inserted between HindIII and PstI sites | This study |
| pABW1-REP-W10NP1 | Km$^r$; 6.7 kb; pABW1 derivative carrying REP module of plasmid pW10NP1 (amplified by PCR with primers LREPW10NP1 and RREPW10NP1) inserted between BamHI and HindIII sites | This study |
| pABW1-REP-W11NP1 | Km$^r$; 6.9 kb; pABW1 derivative carrying REP module of plasmid pW11NP1 (amplified by PCR with primers LREPW11NP1 and RREPW11NP1) inserted between HindIII and PstI sites | This study |
| pABW1-REP-W11NP2 | Km$^r$; 6.3 kb; pABW1 derivative carrying REP module of plasmid pW11NP2 (amplified by PCR with primers LREPW11NP2 and RREPW11NP2) inserted between BamHI and EcoRI sites | This study |
| pBBR-CDF-H6N | Km$^r$; 6.8 kb; pBBR1MSC-2 derivative carrying CDF module of plasmid pH6NP1 (amplified by PCR with primers LcdfH6NP1 and RcdfH6NP1) inserted between BamHI and EcoRI sites | This study |
| pBBR-MER-H6N | Km$^r$; 8.8 kb; pBBR1MSC-2 derivative carrying MER module of plasmid pH6NP1 (amplified by PCR with primers Lmer_H6NP1 and Rmer_H6NP1) inserted between BamHI and KpnI sites of pBBR1MSC-2 | This study |
| pBBR-ZNT-E3S | Km$^r$; 8.5 kb; pBBR1MSC-2 derivative carrying ZNT module of plasmid pE3SP1 (amplified by PCR with primers Lznt_E3SP1 and Rznt_uni) inserted between HindIII and KpnI sites of pBBR1MSC-2 | This study |
| pBBR-ZNT-E10S | Km$^r$; 8.1 kb; pBBR1MSC-2 derivative carrying ZNT module of plasmid pE10SP1 (amplified by PCR with primers Lznt_E10SP1 and Rznt_uni) inserted between HindIII and KpnI sites of pBBR1MSC-2 | This study |
| pBBR-ZNU-E5S | Km$^r$; 7.6 kb; pBBR1MSC-2 derivative carrying ZNU module of plasmid pE5SP1 (amplified by PCR with primers LznuA and RznuA) inserted between BamHI and HindIII sites | This study |
| pBBR-ZNU-E19S | Km$^r$; 7.6 kb; pBBR1MSC-2 derivative carrying ZNU module of plasmid pE19SP1 (amplified by PCR with primers LznuA and RznuA) inserted between BamHI and HindIII sites | This study |

* Natural plasmids of *Polaromonas* spp. identified and characterized in this study are listed in Table 1.

## References:

Bartosik, D., Bialkowska, A., Baj, J., and Wlodarczyk, M. (1997). Construction of mobilizable cloning vectors derived from pBGS18 and their application for analysis of replicator region of a pTAV202 mini-derivative of *Paracoccus versutus* pTAV1 plasmid. *Acta Microbiol. Pol.* 46**,** 387-392.

Kovach, M.E., Phillips, R.W., Elzer, P.H., Roop, R.M., 2nd, and Peterson, K.M. (1994). pBBR1MCS: a broad-host-range cloning vector. *BioTechniques* 16**,** 800-802.
