# Supplementary material for: Plasmids of Psychrotolerant Polaromonas spp. Isolated From Arctic and Antarctic Glaciers – Diversity and Role in Adaptation to Polar Environments
Source: Front Microbiol. 2018 Jun 18;9:1285. doi: 10.3389/fmicb.2018.01285 (PMC6015842; doi:10.3389/fmicb.2018.01285)
Supplement: Supplementary file 3 [file Table_3.PDF]

Supplementary Material

Plasmids of Psychrotolerant *Polaromonas* spp. Isolated from Arctic and Antarctic Glaciers – Diversity and Role in Adaptation to Polar Environments

Anna Ciok[1], Karol Budzik[1], Marek K. Zdanowski[2], Jan Gawor[3], Jakub Grzesiak[2], Przemyslaw Decewicz[1], Robert Gromadka[3], Dariusz Bartosik[1], Lukasz Dziewit[1*]

*** Correspondence:** Dr. Lukasz Dziewit: ldziewit@biol.uw.edu.pl

TABLE S3. Primers used in this study.

Primer	Sequence[a]	Position[b]
LREPE3SP1	gcggaattcTGTCCATGACTGCTGATACG	101,029-101,048 [pE3SP1]
RREPE3SP1	ataggatccCGGCGGTAAACGCAGTAAAG	2,006-1,987 [pE3SP1]
LREPE5SP1	cgcgaattcACGCGGTCTGACTGTGCTTG	65,446-65,465 [pE5SP1]
RREPE5SP1	gtaggatccACCGATTGCGGCGGTAAACG	2,014-1,995 [pE5SP1]
LREPE10SP1	cgcgaattcTGTCCATGACCGCTGATACG	86,240-86,259 [pEclE10SP1]
RREPE10SP1	gtaggatccAACCGATTGCGGCGGTAAAC	2,009-1,990 [pEclE10SP1]
LREPE19SP1	agggatccAGGCCGTAACAGCTGAATTG	18,644-18,663 [pE19SP1]
RREPE19SP1	acctgcagAGTCCCGCAGTTGGAATATC	2,044-2,025 [pE19SP1]
LREPH1NP1	ccgaattcCCGCAACCAGCAACTGAACG	29,344-29,363 [pH1NP1]
RREPH1NP1	aaggatccTCCTGGTGCGCATGAGTTCC	1,728-1,709 [pH1NP1]
LREPH6NP1	gcgaattcCTCGCAGGACTGCATTAGTG	82,485-82,504 [pH6NP1]
RREPH6NP1	atggatccGGCAAAGGACCGCTCGATTC	1,750-1,731 [pH6NP1]
LREPH8NP1	atctgcagGCACTTCGCGCATACATTTC	11,169-11,188 [pH8NP1]
RREPH8NP1	cgaagcttCCAAGGCCTCATGACTTATC	1,508-1,489 [pH8NP1]
LREPH8NP2	gcgaattcCTTCGCCCAAGCACTCACAG	38,017-38,036 [pH8NP2]
RREPH8NP2	atggatccCGCCAGCGGATCATCGAAAG	2,238-2,219 [pH8NP2]
LREPW5NP1	gcgaagcttGTGAGCTTGCCGGTAAAGAG	9,530-9,549 [pW5NP1]
RREPW5NP1	gtaggatccCAGTTCGGTCAACGGATACG	1,634-1,615 [pW5NP1]
LREPW9NP1	atctgcagGCAGTTCGACAAGTCGATTC	1,512-1,531 [pW9NP1]
RREPW9NP1	cgaagcttGCGTGGATAAGCCTAAACAG	2,612-2,593 [pW9NP1]
LREPW10NP1	cgaagcttTGGTGGCCAGGAGAAATTAC	20,697-20,716 [pW10NP1]
RREPW10NP1	atggatccTACGGCCTTGCTGAGATATG	2,106-2,087 [pW10NP1]
LREPW11NP1	atctgcagTCGAGGTGCCTGTCGAAGTG	462-481 [pW11NP1]
RREPW11NP1	cgaagcttTGGAATCGGGCCGACCTTTG	2,907-2,888 [pW11NP1]
LREPW11NP2	gcgaattcATGGTGAGCCAGTGCCTGAG	52,298-52,317 [pW11NP2]
RREPW11NP2	atggatccTGGGCGGTGATTACGGTGTG	1,717-1,698 [pW11NP2]
LcdfH6NP1	gcgaattcCACCAAGTAGGGGTCTGAGG	54,049-54,068 [pH6NP1]
RcdfH6NP1	gaggatccCATTCGCCAAGGCACAGAATG	55,659-55,639 [pH6NP1]
Lmer_H6NP1	ggggtaccAGCGCACGGTTGAGCCTTTG	46,472-46,491 [pH6NP1]
Rmer_H6NP1	atggatccTCCGGTGCCAATGACAACAAG	50,103-50,083 [pH6NP1]
Lznt_E3SP1	cgggtaccCCTTGTCGACTGCACGATATAG	86,765-86,786 [pE3SP1]
Lznt_E10SP1	cgggtaccCCTTGTCGACTGCACGGTATAG	45,710-45,731 [pE10SP1]
Rznt_uni	cgaagcttTCGCTGGATTCCGGCATCTTCG	90,109-90,088 [pE3SP1], 48,650-48,629 [pE10SP1]
LznuA	ttaggatccCCTTACGAACGCTGATAACC	32,337-32,318 [pE5SP1], 11,132-11,151 [pE19SP1]
RznuA	gtcaagcttCTTGTGCGATGGCAATTGAG	29,884-29,903 [pE5SP1], 13,593-13,574 [pE19SP1]
21M13	TGTAAAACGACGGCCAGT	Universal primer
M13 Rev	CAGGAAACAGCTATGACC	Universal primer

[a] Primers are shown in the 5′ to 3′ orientation. Sequence not complementary to the plasmids sequences are shown in lowercase. Introduced restriction sites are underlined.

[b] Position of primers in relation to the particular plasmid. Plasmid name is indicated in square brackets.
