# Supplementary material for: Plasmids of Psychrotolerant Polaromonas spp. Isolated From Arctic and Antarctic Glaciers – Diversity and Role in Adaptation to Polar Environments
Source: Front Microbiol. 2018 Jun 18;9:1285. doi: 10.3389/fmicb.2018.01285 (PMC6015842; doi:10.3389/fmicb.2018.01285)
Supplement: Supplementary file 4 [file Table_4.PDF]

Supplementary Material

Plasmids of Psychrotolerant *Polaromonas* spp. Isolated from Arctic and Antarctic Glaciers – Diversity and Role in Adaptation to Polar Environments

Anna Ciok[1], Karol Budzik[1], Marek K. Zdanowski[2], Jan Gawor[3], Jakub Grzesiak[2], Przemyslaw Decewicz[1], Robert Gromadka[3], Dariusz Bartosik[1], Lukasz Dziewit[1*]

*** Correspondence:** Dr. Lukasz Dziewit: ldziewit@biol.uw.edu.pl

TABLE S4. Genes located within *Polaromonas* plasmids.

Genes located within the plasmid pE3SP1 of *Polaromonas* sp. E3S

Gene	Location	Protein length (aa)	Predicted function
pE3SP1_p001	640 - 1962	440	replication initiation protein
pE3SP1_p002	1959 - 3314	451	DNA helicase, COG0305
pE3SP1_p003	4375 - 5910	511	transposase, IS21 family, COG4584
pE3SP1_p004	5931 - 6704	257	ATPase involved in transposition, IS21 family
pE3SP1_p005	6887 - 7969	360	transposase, IS21 family, COG4584
pE3SP1_p006	7966 - 8751	261	ATPase involved in transposition, IS21 family
pE3SP1_p007	9069 - 9638	189	transposase, IS66 family, partial
pE3SP1_p008	10816 - 10451	121	toxin, Doc family, COG3654
pE3SP1_p009	11582 - 11277	101	antitoxin of addiction system, HigA family
pE3SP1_p010	11887 - 11618	89	toxin of addiction system, HigB family, COG3549
pE3SP1_p011	12230 - 13210	326	transposase, IS5 family, partial
pE3SP1_p012	13533 - 13808	91	hypothetical protein
pE3SP1_p013	15015 - 15530	171	hypothetical protein
pE3SP1_p014	16668 - 15673	331	lipoate synthase LipA, COG0320 [EC:2.8.1.8]
pE3SP1_p015	17412 - 16750	220	Fe-S cluster biogenesis scaffold protein Nfu/NifU
pE3SP1_p016	18652 - 17405	415	membrane protein
pE3SP1_p017	19294 - 18734	186	Fe-S cluster biogenesis scaffold protein Nfu/NifU
pE3SP1_p018	19671 - 19351	106	glutaredoxin-related protein, COG0278
pE3SP1_p019	19933 - 19685	82	transcriptional regulator, BolA superfamily, COG5007
pE3SP1_p020	20419 - 19970	149	Fe-S cluster assembly protein IscU, COG0822
pE3SP1_p021	21672 - 20419	417	cysteine sulfinate desulfinase SufS, COG0520 [EC:2.8.1.7/4.4.1.16]
pE3SP1_p022	22985 - 21669	438	Fe-S cluster assembly protein SufD, COG0719
pE3SP1_p023	23782 - 22982	266	Fe-S cluster assembly ATPase SufC, COG0396

pE3SP1_p024	25215 - 23779	478	Fe-S cluster assembly scaffold protein SufB, COG0719
pE3SP1_p025	25629 - 25279	116	Fe-S cluster assembly iron-binding protein IscA, COG0316
pE3SP1_p026	25904 - 26602	232	transposase, IS6 family, partial, COG3316
pE3SP1_p027	26837 - 26568	89	transcriptional repressor, CopG family
pE3SP1_p028	27518 - 26877	213	partitioning protein, ParA, COG1192
pE3SP1_p029	28164 - 27583	207	serine recombinase, COG1961
pE3SP1_p030	29379 - 28465	304	transcriptional regulator, LysR family, COG0583
pE3SP1_p031	29483 - 30460	325	transcriptional regulator, NmrA family
pE3SP1_p032	31128 - 30514	204	serine recombinase, COG1961
pE3SP1_p033	32407 - 31190	405	transposition auxiliary protein, TniQ
pE3SP1_p034	33312 - 32404	302	transposition auxiliary protein, TniB
pE3SP1_p035	34994 - 33315	559	putative transposase TniA
pE3SP1_p036	35168 - 35461	97	toxin of addiction module, ParE superfamily, COG3657
pE3SP1_p037	35476 - 35781	101	putative antitoxin of addiction module
pE3SP1_p038	36011 - 36286	91	toxin of addiction module, BrnT superfamily, COG2929
pE3SP1_p039	36264 - 36542	92	antitoxin of addiction module, BrnA family, COG3514
pE3SP1_p040	37748 - 37380	122	cytochrome C oxidase
pE3SP1_p041	38974 - 37751	407	periplasmic DMSO/TMAO reductase, COG2041
pE3SP1_p042	40224 - 39637	195	DNA-binding response regulator, NarL/FixJ family, COG2197
pE3SP1_p043	41938 - 40556	460	glucose/arabinose dehydrogenase, COG2133
pE3SP1_p044	42903 - 42295	202	transposase, IS6 family, COG3316
pE3SP1_p045	43924 - 43355	189	hypothetical protein
pE3SP1_p046	44526 - 44789	87	antitoxin of addiction module, RelB/DinJ family, COG3077
pE3SP1_p047	44767 - 45081	104	toxin of addiction module, RelE/StbE family, COG3041
pE3SP1_p048	45800 - 46003	67	toxin of addiction module, ParE superfamily, partial
pE3SP1_p049	46000 - 46281	93	predicted antitoxin, DNA-binding protein with XRE-type HTH domain, COG5606
pE3SP1_p050	47098 - 46406	230	transposase, IS6 family, COG3316
pE3SP1_p051	48316 - 50127	603	signal protein containing EAL and GGDEF domain, COG2200 and COG2199
pE3SP1_p052	52356 - 51583	257	ATPase involved in transposition, IS21 family
pE3SP1_p053	53462 - 52377	361	transposase, IS21 family, partial, COG4584
pE3SP1_p054	53529 - 54611	360	transposase, IS21 family, COG4584
pE3SP1_p055	54608 - 55393	261	ATPase involved in transposition, IS21 family
pE3SP1_p056	56030 - 55434	198	transposase, IS21 family, partial, COG4584
pE3SP1_p057	57341 - 56835	168	acetolactate synthase, small subunit IlvH, COG0440 [EC:2.2.1.6]
pE3SP1_p058	59164 - 57452	570	acetolactate synthase, large subunit IlvB, COG0028 [EC:2.2.1.6]
pE3SP1_p059	59921 - 59226	231	hypothetical protein
pE3SP1_p060	61080 - 59968	370	molybdenum cofactor biosynthesis enzyme MoaA, COG2896
pE3SP1_p061	62800 - 61145	551	oxidoreductase from McbC family
pE3SP1_p062	64707 - 62902	601	uncharacterized conserved protein, COG0397
pE3SP1_p063	65314 - 64712	200	Fe-S cluster biogenesis scaffold protein Nfu/NifU

pE3SP1_p064	66596 - 65343	417	membrane protein
pE3SP1_p065	67049 - 66726	107	rhodanese-related sulfurtransferase, COG0607
pE3SP1_p066	68118 - 67150	322	lipoate synthase LipA, COG0320 [EC:2.8.1.8]
pE3SP1_p067	68728 - 68405	107	protein containing 4Fe-4S dicluster domain
pE3SP1_p068	69322 - 68756	188	GTP cyclohydrolase I, COG0302 [EC:3.5.4.16]
pE3SP1_p069	69622 - 69323	99	transcriptional regulator, BolA superfamily, COG5007
pE3SP1_p070	70211 - 69651	186	Fe-S cluster biogenesis scaffold protein Nfu/NifU
pE3SP1_p071	70594 - 70274	106	glutaredoxin-related protein, COG0278
pE3SP1_p072	70856 - 70611	81	transcriptional regulator, BolA superfamily, COG5007
pE3SP1_p073	71382 - 70897	161	Fe-S cluster formation protein IscU, COG0822
pE3SP1_p074	72650 - 71391	419	cysteine sulfinate desulfinase, SufS, COG0520 [EC:2.8.1.7/4.4.1.16]
pE3SP1_p075	73999 - 72647	450	Fe-S cluster assembly scaffold protein SufD
pE3SP1_p076	74805 - 73996	269	Fe-S cluster assembly ATPase SufC, COG0396
pE3SP1_p077	76247 - 74802	481	Fe-S cluster assembly scaffold protein SufB, COG0719
pE3SP1_p078	76647 - 76279	122	Fe-S cluster assembly iron-binding protein IscA, COG0316
pE3SP1_p079	77009 - 76644	121	Fe-S cluster assembly iron-binding protein IscA, COG0316
pE3SP1_p080	78875 - 77820	351	transposase, IS5 family
pE3SP1_p081	79240 - 79932	230	transposase, IS6 family, COG3316
pE3SP1_p082	80072 - 80764	230	transposase, IS6 family, COG3316
pE3SP1_p083	82309 - 80954	451	NADH dehydrogenase, FAD-containing subunit, COG1252
pE3SP1_p084	83022 - 82399	207	transcriptional regulator, MarR family, COG1846
pE3SP1_p085	83155 - 85113	652	aconitate hydratase [EC:4.2.1.3]
pE3SP1_p086	85138 - 85629	163	sulfite reductase subunit alpha, CysJ, partial
pE3SP1_p087	85626 - 85874	82	sulfite reductase subunit alpha, CysJ, partial
pE3SP1_p088	87050 - 86460	196	transposase, IS6 family, COG3316
pE3SP1_p089	87459 - 89861	800	P-type heavy metal-transporting ATPase, ZntA, COG2217 [EC:3.6.3.54]
pE3SP1_p090	90500 - 91192	230	transposase, IS6 family, COG3316
pE3SP1_p091	92226 - 91924	100	transposase, IS6 family, partial, COG3316
pE3SP1_p092	93253 - 92645	202	transcriptional regulator, MarR family, COG1846
pE3SP1_p093	93797 - 93375	140	transposase, IS3 family, partial, COG2801
pE3SP1_p094	93882 - 94574	230	transposase, IS6 family, COG3316
pE3SP1_p095	94815 - 94498	105	antitoxin of addiction module, RelB
pE3SP1_p096	95203 - 97101	632	tyrosine recombinase
pE3SP1_p097	97140 - 97397	85	plasmid stability protein, COG4691
pE3SP1_p098	97394 - 97813	139	predicted nucleic acid-binding protein, COG1487
pE3SP1_p099	99926 - 98865	353	partitioning protein ParB, COG1475
pE3SP1_p100	101077- 99923	384	partitioning protein ParA, COG1192

Genes located within the plasmid pE5SP1 of *Polaromonas* sp. E5S

Gene	Location	Protein length (aa)	Predicted function
pE5SP1_p001	640 - 1962	440	replication initiation protein
pE5SP1_p002	1959 - 3314	451	DNA helicase, COG0305
pE5SP1_p003	4375 - 5910	511	transposase, IS21 family, COG4584
pE5SP1_p004	5931 - 6704	257	ATPase involved in transposition, IS21 family, COG1484
pE5SP1_p005	6975 - 7520	181	transposase, IS66 family, partial
pE5SP1_p006	7797 - 7549	82	hypothetical protein
pE5SP1_p007	8698 - 8333	121	toxin, Doc family, COG3654
pE5SP1_p008	9464 - 9159	101	antitoxin of addiction module, HigA family, COG3093
pE5SP1_p009	9769 - 9500	89	toxin of addiction module, HigB family, COG3549
pE5SP1_p010	10112 - 10603	163	transposase, IS5 family, partial
pE5SP1_p011	11415 - 11690	91	hypothetical protein
pE5SP1_p012	12332 - 12670	112	hypothetical protein
pE5SP1_p013	12897 - 13412	171	transposase, IS5 family, partial
pE5SP1_p014	14550 - 13555	331	lipoate synthase LipA, COG0320 [EC:2.8.1.8]
pE5SP1_p015	15294 - 14632	220	Fe-S cluster biogenesis scaffold protein Nfu/NifU
pE5SP1_p016	16534 - 15287	415	membrane protein
pE5SP1_p017	17176 - 16616	186	Fe-S cluster biogenesis scaffold protein Nfu/NifU
pE5SP1_p018	17553 - 17233	106	glutaredoxin-related protein, COG0278
pE5SP1_p019	17815 - 17567	82	transcriptional regulator, BolA superfamily, COG5007
pE5SP1_p020	18301 - 17852	149	Fe-S cluster formation protein IscU, COG0822
pE5SP1_p021	19554 - 18301	417	cysteine sulfinate desulfinase, SufS, COG0520 [EC:2.8.1.7/4.4.1.16]
pE5SP1_p022	20867 - 19551	438	Fe-S cluster assembly scaffold protein SufD
pE5SP1_p023	21664 - 20864	266	Fe-S cluster assembly ATPase SufC, COG0396
pE5SP1_p024	23097 - 21661	478	Fe-S cluster assembly scaffold protein SufB, COG0719
pE5SP1_p025	23511 - 23161	116	Fe-S cluster assembly iron-binding protein IscA, COG0316
pE5SP1_p026	23786 - 24484	232	transposase, IS6 family, partial, COG3316
pE5SP1_p027	24719 - 24450	89	transcriptional regulator, CopG family
pE5SP1_p028	25400 - 24759	213	partitioning protein ParA, COG1192
pE5SP1_p029	26046 - 25465	193	serine recombinase, COG1961
pE5SP1_p030	26234 - 26527	97	toxin of addiction system, ParE family, COG3657
pE5SP1_p031	26542 - 26847	101	putative addiction module antidote protein, DNA-binding protein, COG3636
pE5SP1_p032	27263 - 26931	110	transposase, IS6 family, partial, COG3316
pE5SP1_p033	29676 - 27574	700	TonB-dependent outer membrane receptor protein, COG1629
pE5SP1_p034	30981 - 30073	302	Zn-binding component of ABC-type Zn uptake system, ZnuA, COG0803
pE5SP1_p035	32110 - 32268	52	toxin of addiction module, BrnT superfamily, partial, possible frameshift , COG2929
pE5SP1_p036	32265 - 32384	39	toxin of addiction module, BrnT superfamily, partial, possible frameshift , COG2929
pE5SP1_p037	32362 - 32640	92	antitoxin of addiction module, BrnA family, COG3514

pE5SP1_p038	33617 - 33078	179	DNA-binding response regulator, NarL/FixJ family, COG2197
pE5SP1_p039	35381 - 33999	460	glucose/arabinose dehydrogenase, COG2133
pE5SP1_p040	38863 - 35879	994	transposase, Tn*3* family, COG4644
pE5SP1_p041	38984 - 39601	205	serine recombinase, COG1961
pE5SP1_p042	39830 - 40906	358	putative topoisomerase
pE5SP1_p043	41883 - 41086	265	hypothetical protein
pE5SP1_p044	42094 - 42396	100	transposase, IS3 family, COG2963
pE5SP1_p045	43148 - 42666	160	hypothetical protein
pE5SP1_p046	43921 - 43346	191	serine recombinase, COG1961
pE5SP1_p047	44085 - 45029	314	transposase, Tn*3* family, partial, COG4644
pE5SP1_p048	45096 - 47057	653	transposase, Tn*3* family, partial, COG4645
pE5SP1_p049	47094 - 47702	202	conjugal transfer relaxase TraI, partial
pE5SP1_p050	48706 - 48068	212	hypothetical protein
pE5SP1_p051	50405 - 50127	92	antitoxin of addiction module, BrnA family, COG3514
pE5SP1_p052	50478 - 50383	31	toxin of addiction module, BrnT superfamily, partial
pE5SP1_p053	51902 - 52438	178	hypothetical protein with domain of unknown function (DUF4402)
pE5SP1_p054	52911 - 53447	178	hypothetical protein with domain of unknown function (DUF4402)
pE5SP1_p055	53506 - 54357	283	P pilus assembly chaperone protein, COG3121
pE5SP1_p056	54435 - 57125	896	hypothetical protein
pE5SP1_p057	57550 - 57858	102	transposase, partial
pE5SP1_p058	57858 - 58439	193	outer membrane receptor protein, partial, COG1629
pE5SP1_p059	58582 - 59274	230	transposase, IS6 family, COG3316
pE5SP1_p060	59604 - 61502	632	tyrosine recombinase
pE5SP1_p061	61541 - 61798	85	plasmid stability protein, COG4691
pE5SP1_p062	61795 - 62214	139	predicted nucleic acid-binding protein, COG1487
pE5SP1_p063	64326 - 63265	353	partitioning protein ParB, COG1475
pE5SP1_p064	65477 - 64323	384	partitioning protein ParA, COG1192

Genes located within the plasmid pE10SP1 of *Polaromonas* sp. E10S

**Gene**	**Location**	**Protein length (aa)**	**Predicted function**
pE10SP1_p001	634 - 1956	440	replication initiation protein
pE10SP1_p002	1953 - 3308	451	DNA helicase DnaB, COG0305
pE10SP1_p003	3802 - 3386	138	transposase, COG3316
pE10SP1_p004	3795 - 4706	303	Protein of unknown function (DUF1016)
pE10SP1_p005	4703 - 6826	707	DEAD-like helicase
pE10SP1_p006	6819 - 8009	396	putative helicase
pE10SP1_p007	8217 - 8810	197	hypothetical protein
pE10SP1_p008	11640 - 9250	796	uncharacterized protein conserved in bacteria
pE10SP1_p009	12254 - 11685	189	serine recombinase, COG1961
pE10SP1_p010	14381 - 12591	596	uncharacterized protein, COG3472 and COG1479
pE10SP1_p011	14985 - 14707	92	antitoxin of addiction module, BrnA family, COG3514
pE10SP1_p012	15136 - 14963	57	toxin of addiction module, BrnT superfamily, partial, possible frameshift , COG2929
pE10SP1_p013	15237 - 15079	52	toxin of addiction module, BrnT superfamily, partial, possible frameshift , COG2929
pE10SP1_p014	15453 - 15755	100	transposase, IS3 family, partial, COG2963
pE10SP1_p015	16645 - 16025	206	hypothetical protein
pE10SP1_p016	17089 - 16775	104	toxin of addiction module, RelE/StbE family, COG3041
pE10SP1_p017	17330 - 17067	87	antitoxin of addiction module, RelB, COG3077
pE10SP1_p018	17757 - 19139	460	glucose/arabinose dehydrogenase, COG2133
pE10SP1_p019	19473 - 20060	195	DNA-binding response regulator, NarL/FixJ family, COG2197
pE10SP1_p020	20776 - 20498	92	antitoxin of addiction module, BrnA family, COG3514
pE10SP1_p021	20873 - 20754	39	toxin of addiction module, BrnT superfamily, partial, possible frameshift , COG2929
pE10SP1_p022	21028 - 20870	52	toxin of addiction module, BrnT superfamily, partial, possible frameshift , COG2929
pE10SP1_p023	22165 - 23073	302	Zn-binding component of ABC-type Zn uptake system, ZnuA, COG0803
pE10SP1_p024	23178 - 23441	87	hypothetical protein
pE10SP1_p025	23470 - 25572	700	TonB-dependent outer membrane receptor protein, COG1629
pE10SP1_p026	25893 - 26225	142	transposase, IS6 family, partial, COG3316
pE10SP1_p027	26614 - 26309	101	putative antitoxin, HTH domain-containing protein
pE10SP1_p028	26916 - 26629	95	Putative toxin, ParE superfamily, COG3657
pE10SP1_p029	27110 - 27691	193	serine recombinase, COG1961
pE10SP1_p030	27756 - 28397	213	partitioning protein, ParA, COG1192
pE10SP1_p031	29373 - 28672	233	transposase, IS6 family, partial, COG3316
pE10SP1_p032	29649 - 29999	116	Fe-S cluster assembly iron-binding protein IscA, COG0316
pE10SP1_p033	30063 - 31499	478	Fe-S cluster assembly scaffold protein SufB, COG0719
pE10SP1_p034	31496 - 32296	266	Fe-S cluster assembly ATPase SufC, COG0396
pE10SP1_p035	32293 - 33609	438	Fe-S cluster assembly scaffold protein SufD
pE10SP1_p036	33606 - 34859	417	cysteine sulfinate desulfinase, SufS, COG0520 [EC:2.8.1.7/4.4.1.16]
pE10SP1_p037	34859 - 35308	149	Fe-S cluster formation protein IscU, COG0822

pE10SP1_p038	35345 - 35593	82	transcriptional regulator, BolA superfamily, COG5007
pE10SP1_p039	35607 - 35927	106	glutaredoxin-related protein, COG0278
pE10SP1_p040	35984 - 36544	186	Fe-S cluster biogenesis scaffold protein Nfu/NifU
pE10SP1_p041	36626 - 37873	415	membrane protein
pE10SP1_p042	37866 - 38528	220	Fe-S cluster biogenesis scaffold protein Nfu/NifU
pE10SP1_p043	38610 - 39605	331	lipoate synthase LipA, COG0320 [EC:2.8.1.8]
pE10SP1_p044	40581 - 39748	277	transposase, IS5 family, partial
pE10SP1_p045	41098 - 41367	89	toxin of addiction module, HigB family, COG3549
pE10SP1_p046	41403 - 41708	101	antitoxin of addiction module, HigA family
pE10SP1_p047	41995 - 42156	53	hypothetical protein
pE10SP1_p048	42169 - 42534	121	toxin, Doc family, COG3654
pE10SP1_p049	43070 - 43318	82	hypothetical protein
pE10SP1_p050	43955 - 43347	202	transposase, IS66 family, partial
pE10SP1_p051	44876 - 44184	230	transposase, IS6 family, COG3316
pE10SP1_p052	45386 - 44883	167	P-type heavy metal-transporting ATPase, ZntA, partial, COG2217
pE10SP1_p053	45791 - 45405	128	transposase, IS6 family, partial, COG3316
pE10SP1_p054	46000 - 48402	800	P-type heavy metal-transporting ATPase, ZntA, COG2217 COG2217 [EC:3.6.3.54]
pE10SP1_p055	48986 - 49480	164	transposase, IS6 family, partial, COG3316
pE10SP1_p056	49935 - 49573	120	calcineurin-like phosphoesterase, partial
pE10SP1_p057	50096 - 50887	263	ABC-type amino acid transport system, periplasmic component, COG0834
pE10SP1_p058	51036 - 51707	223	ABC-type amino acid transport system, permease component, COG0765
pE10SP1_p059	51713 - 52444	243	ABC-type polar amino acid transport system, ATPase component, COG1126 [EC:3.6.3.-]
pE10SP1_p060	52555 - 53802	415	selenocysteine lyase/systeine desulfurase, COG0520
pE10SP1_p061	53805 - 55217	470	dihydroorotase or related cyclic amidohydrolase, COG0044 [EC:3.5.2.2]
pE10SP1_p062	55450 - 56418	322	DNA-binding transcriptional regulator, MurR/RpiR family, COG1737
pE10SP1_p063	56412 - 56939	175	ureidoglycolate lyase, COG3194 [EC:4.3.2.3]
pE10SP1_p064	58208 - 57066	380	cyanuric acid hydrolase [EC:3.5.2.15]
pE10SP1_p065	58356 - 59426	356	ABC-type Fe3+/spermidine/putrescine transport systems, ATPase component, PotA, COG3842 [EC:3.6.3.31]
pE10SP1_p066	59499 - 60779	426	spermidine/putrescine-binding periplasmic protein, PotD, COG0687
pE10SP1_p067	60802 - 61713	303	ABC-type spermidine/putrescine transport system, permease component I, PotB, COG1176
pE10SP1_p068	61716 - 62603	295	ABC-type spermidine/putrescine transport system, permease component II, PotC, COG1177
pE10SP1_p069	62684 - 63409	241	Asp/Glu/hydantoin racemase, COG4126
pE10SP1_p070	64376 - 63450	308	putative polysaccharide deacetylase
pE10SP1_p071	64893 - 64453	146	hypothetical protein
pE10SP1_p072	66068 - 65229	279	transposase, IS5 family, partial
pE10SP1_p073	66328 - 68202	624	hypothetical protein
pE10SP1_p074	68879 - 69214	111	transposase, IS30 family, partial, COG2826
pE10SP1_p075	71323 - 70715	202	transcriptional regulator, MarR family, COG1846
pE10SP1_p076	72632 - 72297	111	sulfite reductase subunit alpha, partial
pE10SP1_p077	72935 - 72690	82	sulfite reductase subunit alpha, partial

pE10SP1_p078	73426 - 72935	163	sulfite reductase subunit alpha, partial
pE10SP1_p079	75409 - 73451	652	aconitate hydratase [EC:4.2.1.3]
pE10SP1_p080	75545 - 76168	207	transcriptional regulator, MarR family, COG1846
pE10SP1_p081	76258 - 77613	451	NADH dehydrogenase, FAD-containing subunit, COG1252
pE10SP1_p082	78358 - 77744	204	hypothetical protein
pE10SP1_p083	78797 - 78405	130	molybdenum cofactor synthesis protein, partial
pE10SP1_p084	79481 - 79080	133	radical SAM superfamily, partial
pE10SP1_p085	80235 - 79543	230	transposase, IS6 family, COG3316
pE10SP1_p086	80631 - 82529	632	tyrosine recombinase
pE10SP1_p087	82568 - 82825	85	plasmid stability protein, COG4691
pE10SP1_p088	82822 - 83241	139	predicted nucleic acid-binding protein, COG1487
pE10SP1_p089	85137 - 84076	353	partitioning protein ParB, COG1475
pE10SP1_p090	86294 - 85134	386	partitioning protein ParA, COG1192

Genes located within the plasmid pE19SP1 of *Polaromonas* sp. E19S

**Gene**	**Location**	**Protein length (aa)**	**Predicted function**
pE19SP1_p001	662 - 1468	268	replication initiator protein, COG5534
pE19SP1_p002	2013 - 2273	86	hypothetical protein
pE19SP1_p003	2543 - 2295	82	hypothetical protein
pE19SP1_p004	3362 - 3919	185	serine recombinase, COG1961
pE19SP1_p005	5007 - 4549	152	hypothetical protein
pE19SP1_p006	5925 - 5734	63	hypothetical protein
pE19SP1_p007	6985 - 6599	128	hypothetical protein
pE19SP1_p008	7420 - 7106	104	toxin of addiction module, RelE/StbE family
pE19SP1_p009	7661 - 7398	87	antitoxin of addiction module, RelB/DinJ family
pE19SP1_p010	8088 - 9470	460	glucose/arabinose dehydrogenase, COG2133
pE19SP1_p011	9804 - 10391	195	DNA-binding response regulator, NarL/FixJ family, COG2197
pE19SP1_p012	11107 - 10829	92	antitoxin of addiction module, BrnA family, COG3514
pE19SP1_p013	11204 - 11085	39	toxin of addiction module, BrnT superfamily, partial, possible frameshift, COG2929
pE19SP1_p014	11359 - 11201	52	toxin of addiction module, BrnT superfamily, partial, possible frameshift, COG2929
pE19SP1_p015	12496 - 13404	302	ABC-type Zn uptake system ZnuABC, Zn-binding component ZnuA, COG0803
pE19SP1_p016	13509 - 13772	87	hypothetical protein
pE19SP1_p017	13801 - 15903	700	TonB-dependent outer membrane receptor protein, COG1629
pE19SP1_p018	16103 - 16555	150	transposase, IS6 family, partial, COG3316
pE19SP1_p019	16944 - 16639	101	putative antitoxin of addiction module, COG3636
pE19SP1_p020	17252 - 16959	97	toxin of addiction module, ParE superfamily, COG3657
pE19SP1_p021	17440 - 18021	193	serine recombinase, COG1961
pE19SP1_p022	18052 - 18681	209	partitioning protein ParA, COG1192
pE19SP1_p023	18678 - 18920	80	putative partitioning protein ParB

Genes located within the plasmid pH1NP1 of *Polaromonas* sp. H1N

Gene	Location	Protein length (aa)	Predicted function
pH1NP1_p001	11 - 874	287	replication initiation protein
pH1NP1_p002	1605 - 2588	327	NERD nuclease
pH1NP1_p003	3846 - 3418	142	conjugal transfer protein TraD
pH1NP1_p004	4092 - 6230	712	conjugal transfer MobA/MobL relaxase
pH1NP1_p005	6524 - 6339	61	hypothetical protein
pH1NP1_p006	8052 - 8567	171	transposase, IS3 family
pH1NP1_p007	8564 - 9637	357	transposase, IS3 family, COG2801
pH1NP1_p008	10663 - 10292	123	transcriptional regulator, XRE family, COG1396
pH1NP1_p009	10717 - 11103	128	putative peptidase
pH1NP1_p010	11432 - 12010	192	hypothetical protein
pH1NP1_p011	12398 - 12174	74	XerD recombinase, partial, COG4974
pH1NP1_p012	13818 - 12628	396	putative helicase
pH1NP1_p013	15934 - 13811	707	DEAD-like helicase
pH1NP1_p014	16995 - 15934	353	nuclease of restriction endonuclease-like (RecB) superfamily, COG4804
pH1NP1_p015	19781 - 16992	929	DNA methylase, COG1002
pH1NP1_p016	20942 - 19971	323	XerD recombinase, COG4974
pH1NP1_p017	21106 - 21702	198	hypothetical protein
pH1NP1_p018	21705 - 22796	363	transcriptional regulator, XRE family, COG1396
pH1NP1_p019	23217 - 24578	453	hypothetical protein
pH1NP1_p020	24806 - 26329	507	hypothetical protein
pH1NP1_p021	26937 - 27233	98	hypothetical protein
pH1NP1_p022	27338 - 27985	215	hypothetical protein
pH1NP1_p023	28465 - 29163	232	partitioning protein ParA, COG1192
pH1NP1_p024	29165 - 29488	107	predicted partitioning protein ParB

Genes located within the plasmid pH6NP1 of *Polaromonas* sp. H6N

Gene	Location	Protein length (aa)	Predicted function
pH6NP1_p001	463 - 1248	261	replication initiation protein
pH6NP1_p002	1245 - 1538	97	hypothetical protein
pH6NP1_p003	1695 - 2135	146	peptidase S24 LexA-like, COG2932
pH6NP1_p004	2242 - 3549	435	DNA polymerase V, subunit UmuC, COG0389
pH6NP1_p005	3786 - 4094	102	hypothetical protein
pH6NP1_p006	5021 - 5422	133	hypothetical protein
pH6NP1_p007	6627 - 6241	128	DNA-binding response regulator, OmpR family, COG0745
pH6NP1_p008	6733 - 8265	510	transposase, IS21 family, COG4584
pH6NP1_p009	8258 - 9091	277	ATPase involved in transposition, IS21 family, COG1484
pH6NP1_p010	9459 - 9650	63	hypothetical protein
pH6NP1_p011	10219 - 10548	109	transposase, IS6 family, partial, possible frameshift, COG3316
pH6NP1_p012	10458 - 10928	156	transposase, IS6 family, partial, possible frameshift, COG3316
pH6NP1_p013	12171 - 11101	356	NADPH-dependent FMN reductase, COG0655
pH6NP1_p014	12561 - 13367	268	transposase, IS6 family, partial, COG3316
pH6NP1_p015	14628 - 13498	376	two-component sensor histidine kinase, DNA binding domain, COG2214 and Signal transduction histidine kinase, COG0642
pH6NP1_p016	16330 - 14939	463	two-component sensor histidine kinase, Signal transduction histidine kinase, COG0642 and Signal receiver domain
pH6NP1_p017	17147 - 16728	139	sensor of blue light, FAD-binding domain
pH6NP1_p018	18175 - 17420	251	tyrosine recombinase
pH6NP1_p019	19829 - 18696	377	two-component sensor histidine kinase, Signal transduction histidine kinase, COG0642
pH6NP1_p020	20032 - 20301	89	hypothetical protein
pH6NP1_p021	21126 - 21716	196	chemotaxis response regulator protein-glutamate methylesterase, CheB, partial
pH6NP1_p022	22437 - 22859	140	hypothetical protein
pH6NP1_p023	24510 - 23848	220	transposase, IS3 family, partial, COG2801
pH6NP1_p024	25945 - 25325	206	serine recombinase, COG1961
pH6NP1_p025	26832 - 27941	369	tyrosine recombinase
pH6NP1_p026	29635 - 28919	238	metallophosphatase
pH6NP1_p027	30109 - 31470	453	transposase, IS1380 family
pH6NP1_p028	32328 - 34622	764	catalase-peroxidase, COG0376 [EC:1.11.1.21]
pH6NP1_p029	34994 - 35617	207	tyrosine recombinase
pH6NP1_p030	36006 - 36737	243	NADP-dependent 3-hydroxy acid dehydrogenase, COG4221
pH6NP1_p031	38062 - 37172	296	metallophosphatase
pH6NP1_p032	38914 - 38534	126	hypothetical protein
pH6NP1_p033	39427 - 39011	138	hypothetical protein
pH6NP1_p034	40582 - 39530	350	ATP-dependent Lon protease
pH6NP1_p035	41035 - 41508	157	transposase, IS630 family
pH6NP1_p036	41505 - 42167	220	transposase, IS630 family
pH6NP1_p037	42243 - 43580	445	transposase, IS1182 family, COG3666

pH6NP1_p038	45174 - 45491	105	transcriptional regulator, XRE family, partial, COG1396
pH6NP1_p039	46503 - 46084	139	DNA/RNA non-specific endonuclease
pH6NP1_p040	47004 - 46549	151	DNA-binding transcriptional regulator, MerR family, COG0789
pH6NP1_p041	47101 - 47427	108	mercuric transport protein, integral membrane component, MerT
pH6NP1_p042	47441 - 47716	91	mercuric transport protein, periplasmic component, MerP
pH6NP1_p043	47746 - 48153	135	mercuric transport protein, MerC
pH6NP1_p044	48203 - 49888	561	mercuric reductase, MerA [EC:1.16.1.1]
pH6NP1_p045	50065 - 51414	449	pyruvate/2-oxoglutarate dehydrogenase complex, dihydrolipoamide dehydrogenase (E3) component or related enzyme, COG1249
pH6NP1_p046	53241 - 52162	359	predicted nuclease of restriction endonuclease-like (RecB) superfamily, DUF1016 family, COG4804
pH6NP1_p047	55263 - 54340	307	divalent metal cation transporter, CDF family, COG0053
pH6NP1_p048	56690 - 56457	77	hypothetical protein
pH6NP1_p049	59004 - 57037	655	PAS domain-containing signal transduction protein, COG2202
pH6NP1_p050	59047 - 59520	157	transposase, IS630 family
pH6NP1_p051	59517 - 60179	220	transposase, IS630 family
pH6NP1_p052	61751 - 60174	525	signal transduction protein containing GAF domain
pH6NP1_p053	62137 - 63153	338	transposase, IS110 family, COG3547
pH6NP1_p054	63678 - 64955	425	MntH transporter, COG1914
pH6NP1_p055	65780 - 65460	106	REC (receiver) domain-containing CheY chemotaxis protein , partial, COG0784
pH6NP1_p056	65907 - 66695	262	pimeloyl-ACP methyl ester carboxylesterase, COG0596
pH6NP1_p057	67037 - 68083	348	acetyl esterase/lipase, COG0657
pH6NP1_p058	68725 - 67910	271	transposase, IS701 family, partial
pH6NP1_p059	70316 - 68895	473	transposase, IS4 family
pH6NP1_p060	71513 - 70458	351	transposase, IS701 family
pH6NP1_p061	72332 - 71835	165	hypothetical protein
pH6NP1_p062	73973 - 72345	542	hypothetical protein
pH6NP1_p063	75463 - 74447	338	catalase, COG0753 [EC:1.11.1.6]
pH6NP1_p064	75616 - 75939	107	hypothetical protein
pH6NP1_p065	78361 - 75902	819	response regulator
pH6NP1_p066	78665 - 79324	219	hypothetical protein
pH6NP1_p067	79508 - 81346	612	tyrosine recombinase
pH6NP1_p068	81587 - 82045	152	partitioning protein ParA, partial, possible frameshift, COG1192
pH6NP1_p069	81991 - 82218	75	partitioning protein ParA, partial, possible frameshift, COG1192
pH6NP1_p070	82249 - 82545	98	putative partitioning protein ParB

Genes located within the plasmid pH8NP1 of *Polaromonas* sp. H8N

Gene	Location	Protein legth (aa)	Predicted function
pH8NP1_orf001	255 - 1124	289	replication initiation protein
pH8NP1_orf002	1448 - 1780	110	hypothetical protein
pH8NP1_orf003	1777 - 2439	220	partitioning protein ParA, COG1192
pH8NP1_orf004	2436 - 2690	84	putative partitioning protein ParB
pH8NP1_orf005	2829 - 3224	131	hypothetical protein
pH8NP1_orf006	4024 - 4710	228	serine recombinase, COG1961
pH8NP1_orf007	4948 - 5607	219	protein of unknown function (DUF3102)
pH8NP1_orf008	5669 - 6049	126	hypothetical protein
pH8NP1_orf009	6095 - 7600	501	hypothetical protein
pH8NP1_orf010	7883 - 8497	204	hypothetical protein
pH8NP1_orf011	8515 - 8700	61	hypothetical protein
pH8NP1_orf012	8704 - 8922	72	hypothetical protein
pH8NP1_orf013	10078 - 9656	140	toxin, PemK superfamily, COG3692
pH8NP1_orf014	11215 - 10112	367	hypothetical protein

Genes located within the plasmid pH8NP2 of *Polaromonas* sp. H8N

**Gene**	**Location**	**Protein length (aa)**	**Predicted function**
pH8NP2_p001	38 - 1090	350	replication initiation protein, COG5534
pH8NP2_p002	2134 - 2424	96	toxin of addiction module, BrnT superfamily, COG2929
pH8NP2_p003	2411 - 2713	100	antitoxin of addiction module, BrnA family, COG3514
pH8NP2_p004	3425 - 4924	499	transposase, IS21 family, COG4584
pH8NP2_p005	4973 - 5737	254	ATPase involved in transposition, IS21 family, COG1484
pH8NP2_p006	7358 - 6978	126	putative acetyltransferase, partial
pH8NP2_p007	7756 - 7478	92	conserved protein, DUF1778 family, COG4453
pH8NP2_p008	8412 - 7900	170	hypothetical protein
pH8NP2_p009	9446 - 8763	227	hypothetical protein
pH8NP2_p010	9909 - 9466	147	protein involved in binding peptidoglycan
pH8NP2_p011	10176 - 10487	103	putative conjugal transfer relaxosome component TraJ
pH8NP2_p012	10484 - 13210	908	conjugal transfer relaxase TraI
pH8NP2_p013	14090 - 13905	61	hypothetical protein
pH8NP2_p014	14217 - 15428	403	ATP-dependent Lon protease, COG0466
pH8NP2_p015	16508 - 15435	357	transposase, IS3 family, COG2801
pH8NP2_p016	17020 - 16505	171	transposase, IS3 family
pH8NP2_p017	17098 - 17349	83	hypothetical protein
pH8NP2_p018	17537 - 17737	66	protein of unknown function (DUF3644)
pH8NP2_p019	18108 - 19883	591	uncharacterized conserved protein, COG1479
pH8NP2_p020	20318 - 20584	88	antitoxin of addiction module, HigA family
pH8NP2_p021	21352 - 20669	227	DNA/RNA endonuclease, COG1864
pH8NP2_p022	21797 - 25525	1242	hypothetical protein
pH8NP2_p023	26109 - 27068	319	tyrosine recombinase
pH8NP2_p024	28507 - 27458	349	hypothetical protein
pH8NP2_p025	29060 - 28509	183	hypothetical protein
pH8NP2_p026	29449 - 29060	129	protein with TIR-like domain (DUF1863)
pH8NP2_p027	32614 - 29522	1030	type I site-specific restriction-modification system subunit R, COG0610
pH8NP2_p028	33003 - 32611	130	uncharacterized conserved protein, COG3755
pH8NP2_p029	34105 - 33032	357	protein of unknown function (DUF1016)
pH8NP2_p030	35469 - 34102	455	type I restriction endonuclease S subunit, COG0732
pH8NP2_p031	37012 - 35459	517	type I restriction-modification system, DNA methylase subunit, COG0286
pH8NP2_p032	37490 - 38128	212	partitioning protein ParA, COG1192
pH8NP2_p033	38119 - 38325	68	putative partitioning protein ParB

Genes located within the plasmid pW5NP1 of *Polaromonas* sp. W5N

**Gene**	**Location**	**Protein length (aa)**	**Predicted function**
pW5NP1_p001	555 - 1139	194	replication initiation protein, RepL
pW5NP1_p002	1674 - 1366	102	very-short-patch mismatch repair endonuclease, COG3727
pW5NP1_p003	2368 - 1661	235	nuclease
pW5NP1_p004	2537 - 3151	204	partitioning protein ParA, COG1192
pW5NP1_p005	3467 - 4606	379	DNA-cytosine methyltransferase, COG0270
pW5NP1_p006	4625 - 4930	101	hypothetical protein
pW5NP1_p007	5739 - 5215	174	hypothetical protein
pW5NP1_p008	6017 - 6679	220	hypothetical protein
pW5NP1_p009	7184 - 6690	164	putative coupling protein, TraD
pW5NP1_p010	7399 - 9573	724	conjugal transfer MobA/MobL relaxase

Genes located within the plasmid pW9NP1 of *Polaromonas* sp. W9N

**Gene**	**Location**	**Protein length (aa)**	**Predicted function**
pW9NP1_p001	88 - 1653	521	hypothetical protein
pW9NP1_p002	1637 - 2497	286	replication initiation protein
pW9NP1_p003	2793 - 3122	109	hypothetical protein
pW9NP1_p004	3125 - 3787	220	partitioning protein ParA, COG1192
pW9NP1_p005	3816 - 4043	75	partitioning protein ParG
pW9NP1_p006	4047 - 4655	202	hypothetical protein
pW9NP1_p007	4582 - 4800	72	hypothetical protein
pW9NP1_p008	5361 - 5696	336	HNH endonuclease
pW9NP1_p009	5821 - 6492	223	serine recombinase, COG1961
pW9NP1_p010	6522 - 6761	79	hypothetical protein
pW9NP1_p011	6843 - 7205	120	hypothetical protein

Genes located within the plasmid pW10NP1 of *Polaromonas* sp. W10N

**Gene**	**Location**	**Protein length (aa)**	**Predicted function**
pW10NP1_p001	72 - 1043	323	replication initiation protein, COG5534
pW10NP1_p002	2010 - 2573	187	hypothetical protein
pW10NP1_p003	2582 - 2851	89	hypothetical protein
pW10NP1_p004	2848 - 3279	143	preprotein translocase subunit SecB, COG1952
pW10NP1_p005	3670 - 8640	1656	helicase, COG4889
pW10NP1_p006	8654 - 9229	191	protein with putative zinc- or iron-chelating domain
pW10NP1_p007	9604 - 9395	69	putative partitioning protein ParB
pW10NP1_p008	10246 - 9617	209	partitioning protein ParA, COG1192
pW10NP1_p009	11216 - 10419	265	hypothetical protein
pW10NP1_p010	11917 - 11213	234	hypothetical protein
pW10NP1_p011	12336 - 11914	140	hypothetical protein
pW10NP1_p012	12690 - 13001	103	putative conjugal transfer relaxosome component TraJ
pW10NP1_p013	13088 - 15715	875	conjugal transfer relaxase TraI
pW10NP1_p014	15921 - 16244	107	hypothetical protein
pW10NP1_p015	17637 - 16810	275	SAM-dependent methyltransferase, COG0500
pW10NP1_p016	18066 - 19061	331	radical SAM superfamily enzyme, MoaA/NifB/PqqE/SkfB family, COG0535
pW10NP1_p017	19330 - 19899	189	serine recombinase, COG1961
pW10NP1_p018	20068 - 20526	152	putative antitoxin
pW10NP1_p019	20510 - 20809	99	toxin of addiction module, ParE superfamily, COG3668

Genes located within the plasmid pW11NP1 of *Polaromonas* sp. W11N

Gene	Location	Protein length (aa)	Predicted function
pW11NP1_p001	1178 - 2407	409	replication initiation protein
pW11NP1_p002	2737 - 3378	213	putative recombinase

Genes located within the plasmid pW11NP2 of *Polaromonas* sp. W11N

Gene	Location	Protein length (aa)	Predicted function
pW11NP2_p001	508 - 1521	337	replication initiation protein
pW11NP2_p002	2071 - 1535	178	DNA helicase, COG1199
pW11NP2_p003	2594 - 3688	364	partitioning protein ParB, COG1475
pW11NP2_p004	4661 - 4176	161	hypothetical protein
pW11NP2_p005	4685 - 4990	101	transposase, IS3 family, COG2963
pW11NP2_p006	4987 - 5895	302	transposase, IS701 family, partial, COG2801
pW11NP2_p007	5955 - 6410	151	hypothetical protein
pW11NP2_p008	6600 - 7091	163	transposase, IS701 family, partial
pW11NP2_p009	7543 - 7133	136	hypothetical protein
pW11NP2_p010	8237 - 7554	227	hypothetical protein
pW11NP2_p011	9191 - 8721	156	hypothetical protein
pW11NP2_p012	9203 - 10060	285	glucose/arabinose dehydrogenase, COG2133
pW11NP2_p013	10882 - 10013	289	transposase, IS3 family, possible frameshift, COG2801
pW11NP2_p014	11121 - 10879	80	transposase, IS3 family, possible frameshift, COG2963
pW11NP2_p015	11267 - 11815	182	transposase, IS5 family, partial
pW11NP2_p016	13354 - 12413	313	periplasmic component of ABC-type branched-chain amino acid transport system, COG0683
pW11NP2_p017	15317 - 13692	541	fatty acyl-CoA synthetase, COG0318 [EC:6.2.1.44]
pW11NP2_p018	16088 - 15360	242	transcriptional regulator, GntR family, COG1802
pW11NP2_p019	17490 - 16657	277	transcriptional regulator, IclR family, COG1414
pW11NP2_p020	17581 - 18747	388	acyl-CoA dehydrogenase, COG1960
pW11NP2_p021	18744 - 19913	389	acyl-CoA dehydrogenase, COG1960
pW11NP2_p022	19930 - 20901	323	tripartite-type tricarboxylate transporter family receptor, COG3181
pW11NP2_p023	20901 - 22109	402	acyl-CoA transferase, COG1804
pW11NP2_p024	22736 - 22206	176	transposase, IS5 family, partial, COG3293
pW11NP2_p025	23506 - 22844	220	transposase, IS630 family, partial, possible frameshift
pW11NP2_p026	23976 - 23503	157	transposase, IS630 family, partial, possible frameshift
pW11NP2_p027	24956 - 23874	360	XerC recombinase, COG4973
pW11NP2_p028	25717 - 25295	140	predicted nucleic acid-binding protein, contains PIN domain, COG1487
pW11NP2_p029	25986 - 25726	86	antitoxin, Phd_YefM family
pW11NP2_p030	26193 - 26591	132	ssDNA binding protein
pW11NP2_p031	26797 - 27192	131	calcium-binding protein
pW11NP2_p032	27570 - 29033	487	transposase, IS21 family
pW11NP2_p033	29030 - 29830	266	ATPase involved in transposition, IS21 family, COG1484
pW11NP2_p034	30920 - 31303	127	DNA-binding protein H-NS, COG2916
pW11NP2_p035	32247 - 31624	207	glutathione S-transferase, COG0625 [EC:2.5.1.18]
pW11NP2_p036	33409 - 32522	295	threonine/homoserine efflux transporter RhtA, COG5006
pW11NP2_p037	33704 - 35044	446	toxin HipA, COG3550

pW11NP2_p038	35054 - 35383	109	Putative antitoxin, transcriptional regulator, XRE family
pW11NP2_p039	36989 - 35595	464	transposase, ISNCY family
pW11NP2_p040	38918 - 37161	585	DEAD-like helicase
pW11NP2_p041	39487 - 39158	109	hypothetical protein
pW11NP2_p042	39650 - 39967	105	transcriptional regulator of competence genes, TfoX/Sxy family, COG3070
pW11NP2_p043	40065 - 40472	135	ssDNA binding protein
pW11NP2_p044	41677 - 41027	216	hypothetical protein
pW11NP2_p045	42234 - 41674	186	transcriptional regulator, MerR superfamily
pW11NP2_p046	43665 - 42592	357	transposase, IS3 family, partial, possible frameshift, COG2801
pW11NP2_p047	44186 - 43662	174	transposase, IS3 family, partial, possible frameshift,
pW11NP2_p048	44472 - 44945	157	transposase, IS630 family, partial, possible frameshift
pW11NP2_p049	44942 - 45604	220	transposase, IS630 family, partial, possible frameshift
pW11NP2_p050	46608 - 47306	232	partitioning protein ParA, COG1192
pW11NP2_p051	47303 - 48307	334	partitioning protein ParB, COG1475
pW11NP2_p052	48838 - 49335	165	transposase, IS630 family, partial
pW11NP2_p053	49431 - 50924	497	transposase, IS21 family
pW11NP2_p054	50921 - 51721	266	ATPase involved in transposition, IS21 family, COG1484
pW11NP2_p055	51806 - 52468	220	transposase, IS630 family, partial
