# Supplementary material for: Plasmids of Psychrotolerant Polaromonas spp. Isolated From Arctic and Antarctic Glaciers – Diversity and Role in Adaptation to Polar Environments
Source: Front Microbiol. 2018 Jun 18;9:1285. doi: 10.3389/fmicb.2018.01285 (PMC6015842; doi:10.3389/fmicb.2018.01285)
Supplement: Supplementary file 5 [file Table_5.PDF]

Supplementary Material

Plasmids of Psychrotolerant *Polaromonas* spp. Isolated from Arctic and Antarctic Glaciers – Diversity and Role in Adaptation to Polar Environments

Anna Ciok[1], Karol Budzik[1], Marek K. Zdanowski[2], Jan Gawor[3], Jakub Grzesiak[2], Przemyslaw Decewicz[1], Robert Gromadka[3], Dariusz Bartosik[1], Lukasz Dziewit[1*]

*** Correspondence:** Dr. Lukasz Dziewit: ldziewit@biol.uw.edu.pl

TABLE S5. Sequences of putative origins of replication (*oriV*) of *Polaromonas* plasmids.

Plasmid name	*rep* gene (coordinates)	Sequences and coordinates of putative iterons (DRs) of putative *oriV* [a]	Sequences and coordinates of putative panlidromic sequences of *oriV* [a]
pE3SP1	*pE3SP1_p001* (640 – 1,962)	DR1.1:360-GTCCGTATATTTCCC-374<br>DR1.2:471-GTCCTTGTTTTTCCC-485<br>DR1.3:515-GTCCTACTTTTTCCC-529<br>DR1.4:559-GTCCAGCAATTTACC-573<br><br>Consensus: GTCCDDVWWTTTMCC	617-TGTCCTAAACGTTTAGGACA-636
pE5SP1	*pE5SP1_p001* (640 – 1,962)	DR1.1:360-GTCCGTATATTTCCC-374<br>DR1.2:471-GTCCTTGTTTTTCCC-485<br>DR1.3:515-GTCCTACTTTTTCCC-529<br>DR1.4:559-GTCCAGCAATTTACC-573<br><br>Consensus: GTCCDDVWWTTTMCC	617-TGTCCTAAACGTTTAGGACA-636
pE10SP1	*pE10SP1_p001* (634 – 1,956)	DR1.1:354-GACCGTATATTTCCC-368<br>DR1.2:465-GTCCTTGTTTTTCCC-479<br>DR1.3:509-GTCCTACTTTTTCCC-523<br>DR1.4:553-GTCCAGCAATTTACC-567<br><br>Consensus: GWCCDDVWWTTTMCC	611-TGTCCTAAACGTTTAGGACA-630
pE19SP1	*pE19SP1_p001* (662 – 1,468)	DR1.1:1472-ACGTCATTTCACCCACCTTTTTCTGTGGATAGGC-1505<br>DR1.2:1506-ACGTCATTTCACCCACCTTTTTCTGTGGATAGGC-1539<br>DR1.3:1540-ACGTCATTTCACCCACCTTTT-CTGTGGATAGGC-1572<br><br>Consensus:ACGTCATTTCACCCACCTTTT(T)CTGTGGATAGGC<br><br>DR2.1:1574-CGTCATTTCACCCACC-1589<br>DR2.2:1597-CGTCATTTCACCCACC-1612<br><br>Consensus:CGTCATTTCACCCACC	Not found.
pH1NP1	*pH1NP1_p001* (11 – 874)	DR1.1:878-GGCACGGCATTCTGCTCCC-896<br>DR1.2:967-GGCACGGCATTGCGCTCCC-985<br>DR1.3:996-GGCACGGCATTCTGCTCCC-1014<br>DR1.4:1018-GGCACGGCATTGCGCTCCC-1036<br>DR1.5:1040-GGCACGGCATTCTGCTCCC-1058<br>DR1.6:1062-GGCACGGCATTCTGCTCCC-1080<br>DR1.7:1084-GGCACGGCATTCTGCTCCC-1102<br><br>Consensus: GGCACGGCATTSYGCTCCC	Not found.

pH6NP1	*pH6NP1_p001*<br>(463 – 1,248)	DR1.1:177-CGGTGTTGCACTA-189<br>DR1.2:199-CGGTGTTGGACTA-211<br>DR1.3:222-CGGTCATAAACTA-234<br>DR1.4:253-CGGTGTTGCACTA-265<br>DR1.5:276-CGGTCTTGGACTA-288<br>DR1.6:299-CAGCTTTGGACTA-311<br>DR1.7:325-CGGCGTTGAACTA-337<br>DR1.8:346-CGGTCTTGGACTG-358<br>DR1.9:367-CGGTCTTGCACTA-379<br><br>Consensus: CRGYBWTRVACTR	Not found.
pH8NP1	*pH8NP1_p001*<br>(255 – 1,124)	DR1.1:1129-CACGCATAAATGGTCGCGCC-1148<br>DR1.2:1231-CACGCATAAATGGTCGCGCC-1250<br>DR1.3:1264-CACGCATAAATGGTCGCGGG-1283<br>DR1.4:1286-CACGCATAAATGGTCGCGCC-1305<br><br>Consensus: CACGCATAAATGGTCGCGSS	Not found.
pH8NP2	*pH8NP2_p001*<br>(38 – 1,090)	DR1.1:1104-ACCGGCTATCGCGGATG-1120<br>DR1.2:1126-ACCGGCTATCGCGGATG-1142<br>DR1.3:1148-ACCGGCTATCGCGGATG-1164<br>DR1.4:1302-ACCGGCTATCGCGGATG-1318<br>DR1.5:1342-ACCGGCTATCGCGGATG-1358<br>DR1.6:1364-ACCGGCTATCGCGGATG-1380<br>DR1.7:1387-ACCGGCTATCGCGGATG-1403<br>DR1.8:1410-TCCGGCTATCGCGGATG-1426<br><br>Consensus: WCCGGCTATCGCGGATG	Not found.
pW5NP1	*pW5NP1_p001*<br>(555 – 1,139)	DR1.1:439-AGACAA-444<br>DR1.2:535-AGACAA-540<br><br>Consensus: AGACAA<br><br>DR2.1:448-TGTCCTCTCGAAAGACAA-465<br>DR2.2:469-TGTCCGCTCGAAAGACAA-486<br><br>Consensus: TGTCCKCTCGAAAGACAA	Not found.
pW9NP1	*pW9NP1_p001*<br>(1,637 – 2,497)	DR1.1:2575-CACGCATTAACGGTCGCG-2592<br>DR1.2:2608-CACGCATTAACGGTCGCG-2625<br>DR1.3:2630-CACGCATTAACGGTCGCG-2647<br><br>Consensus: CACGCATTAACGGTCGAG	Not found.
pW10NP1	*pW10NP1_p001*<br>(72 – 1,043)	DR1.1:1083-ACGTACTTTCACCTGCA-1099<br>DR1.2:1116-ACGTACTTTCACCTGCA-1132<br>DR1.3:1149-ACGTACTTTCACCTGCA-1165<br>DR1.4:1171-ACGTACTTTTACCTGCA-1187<br><br>Consensus: ACGTACTTTYACCTGCA	Not found.
pW11NP1	*pW11NP1_p001*<br>(1,178 – 2,407)	Not found.	Not found.
pW11NP2	*pW11NP2_p001*<br>(508 – 1,521)	DR1.1:182-CTACTAGCGGTATGACGTATCG-203<br>DR1.2:204-CTAGTAGCGATGTGACGTATCG-225<br>DR1.3:226-CTATTAGCGTAAAGATGTACCG-247<br>DR1.4:248-CTATTAGCGGTACATCATCGAA-269<br><br>Consensus: CTABTAGCGDWRHRWYRTMBMR	Not found.

[a] Sequences shown in the 5′ to 3′ orientation.
