# Supplementary material for: Plasmids of Psychrotolerant Polaromonas spp. Isolated From Arctic and Antarctic Glaciers – Diversity and Role in Adaptation to Polar Environments
Source: Front Microbiol. 2018 Jun 18;9:1285. doi: 10.3389/fmicb.2018.01285 (PMC6015842; doi:10.3389/fmicb.2018.01285)
Supplement: Supplementary file 6 [file Table_6.PDF]

Supplementary Material

Plasmids of Psychrotolerant *Polaromonas* spp. Isolated from Arctic and Antarctic Glaciers – Diversity and Role in Adaptation to Polar Environments

Anna Ciok[1], Karol Budzik[1], Marek K. Zdanowski[2], Jan Gawor[3], Jakub Grzesiak[2], Przemyslaw Decewicz[1], Robert Gromadka[3], Dariusz Bartosik[1], Lukasz Dziewit[1*]

*** Correspondence:** Dr. Lukasz Dziewit: ldziewit@biol.uw.edu.pl

TABLE S6. Partitioning systems (including sequences of predicted partitioning sites – *parS*) identified within *Polaromonas* plasmids.

**Plasmid name**	***par* genes (coordinates)**	**Sequences and coordinates of putative direct repeats (DR) of *parS* site [a]**	**Homologous PAR system (found based on best BLASTP hit to the ParA protein)**
pE3SP1	*pE3SP1_p100-p099* (101,077 – 98,865)	DR2.1:98534-CCATTAAATTCAACGCCG-98517<br>DR2.2:98758-CCATTAAATTCAACGCCG-98741<br><br>Consensus: CCATTAAATTCAACGCCG	SAMN05444679_13612-SAMN05444679_13611 of *Variovorax* sp. CF079 [GenBank: FMZU01000036]
pE5SP1	*pE5SP1_p064-p063* (65,477 – 63,265)	DR2.1:63158-CCATTAAATTCAACGCCG-63141<br>DR2.2:63935-CCATTAAATTCAACGCCG-62918<br><br>Consensus: CCATTAAATTCAACGCCG	SAMN05444679_13612-SAMN05444679_13611 of *Variovorax* sp. CF079 [GenBank: FMZU01000036]
pE10SP1	*pE10SP1_p090-p089* 86,294 – 84,076)	DR2.1:83968-CCATTAAATTCAACGCCG-83951<br><br>Sequence designated based on identity with corresponding *parS* site present in highly similar plasmids pE3SP1 and pE5SP1. Lack of repeated sequences.	SAMN05444679_13612-SAMN05444679_13611 of *Variovorax* sp. CF079 [GenBank: FMZU01000036]
pE19SP1	*pE19SP1_p022-p023* (18,052 – 18,920)	Not found.	Aam_202_004-Aam_202_003 of *Acidocella aminolytica* DSM 11237 [GenBank: BANC01000196]
pH1NP1	*pH1NP1_p024-p025* (28,465 – 29,488)	DR2.1:28377-AAGTAGCTA-28385<br>DR2.2:28388-AAGTAGCTA-28396<br>DR2.3:28399-TGGTAGCTT-28407<br>DR2.4:28417-AGATAGCTA-28425<br>DR2.5:28436-TGGTAGCTA-28444<br><br>Consensus: WRRTAGCTW	L288_18190-L288_18195 of *Sphingobium quisquiliarum* P25 [GenBank: ATHO01000158]
pH6NP1	*pH6NP1_p068-p069-p070** (81,587 – 82,545)	DR2.1:81470-AGAAATACTTA-81480<br>DR2.2:81482-GTATTTACGTA-81492<br>DR2.3:81498-GTAAATACGTA-81508<br>DR2.4:81514-GTAAATAATTT-81524<br>DR2.5:81538-TTACATGATTT-81548<br><br>Consensus: DKAHWTRMKTW	AVS7_01546-AVS7_01547 of *Acidovorax* sp. MR-S7 [GenBank: DF238910]
pH8NP1	*pH8NP1_p003-p004* (1,777 – 2,690)	DR2.1:1691-CAGAACTT-1698<br>DR2.2:1752-CAGAACTT-1759<br><br>Consensus: CAGAACTT	BWK72_20690-BWK72_20685 of *Rhodoferax ferrireducens* isolate A7 [GenBank: MTEI01000038]

pH8NP2	*pH8NP2_p032-p033*<br>(37,490 – 38,325)	DR2.1:37402-ATACAC-37407<br>DR2.2:37410-ATACAC-37415<br>DR2.3:37418-ATACAC-37423<br>DR2.4:37426-ATACAC-37431<br>DR2.5:37434-ATACAC-37439<br>DR2.6:37466-ATACAC-37471<br>DR2.7:37482-ATACAC-37487<br><br>Consensus: ATACAC	BLL52_3466-BLL52_3465 of *Rhodoferax antarcticus* ANT.BR<br>[GenBank: MSYM01000017]
pW5NP1	*pW5NP1_p004*<br>(2,537 – 3,151)<br><br>(lack of ParB)	DR3.1:2515-TTGCTACCA-2520<br>DR3.2:2528-TTGCTACCA-2536<br><br>Consensus: TTGCTACCA	CJ014_25880 of *Pleomorphomonas* sp. SVCO-16 [GenBank: NQVN01000037]
pW9NP1	*pW9NP1_p004-p005*<br>(3,125 – 4,043)	DR2.1:4047-ATGGCCACC-4055<br>DR2.2:4122-ATGGCCACC-4130<br><br>Consensus: ATGGCCACC	BWK72_20690-BWK72_20685 of *Rhodoferax ferrireducens* isolate A7<br>[GenBank: MTEI01000038]
pW10NP1	*pW10NP1_p008-p007*<br>(10,246 – 9,395)	DR2.1:10276-TTTACATA-10269<br>DR2.2:10268-TTTACATA-10261<br><br>Consensus: TTTACATA	Pnap_4996-Pnap_4995 of plasmid pPNAP07 of *Polaromonas naphthalenivorans* CJ2<br>[GenBank: CP000536]
pW11NP1	Not found.	-	-
pW11NP2	*pW11NP2_050-p051*<br>(46,608 – 48,307)	DR2.1:46506-AATATTAG-46513<br>DR2.2:46514-AATGTTAA-46521<br>DR2.3:46527-AATAGTTC-46534<br>DR2.4:46544-AATATTAG-46551<br>DR2.5:46589-AACATTGA-46596<br><br>Consensus: AAYRKTDV	Pnap_4472-Pnap_4471 of plasmid pPNAP02 of *Polaromonas naphthalenivorans* CJ2<br>[GenBank: CP000531]

[a] Sequences shown in the 5′ to 3′ orientation.

* Possible frameshift mutation allows full-length transcription of the *parA* mRNA.
