# Supplementary material for: Plasmids of Psychrotolerant Polaromonas spp. Isolated From Arctic and Antarctic Glaciers – Diversity and Role in Adaptation to Polar Environments
Source: Front Microbiol. 2018 Jun 18;9:1285. doi: 10.3389/fmicb.2018.01285 (PMC6015842; doi:10.3389/fmicb.2018.01285)
Supplement: Supplementary file 7 [file Table_7.PDF]

Supplementary Material

Plasmids of Psychrotolerant *Polaromonas* spp. Isolated from Arctic and Antarctic Glaciers – Diversity and Role in Adaptation to Polar Environments

Anna Ciok[1], Karol Budzik[1], Marek K. Zdanowski[2], Jan Gawor[3], Jakub Grzesiak[2], Przemyslaw Decewicz[1], Robert Gromadka[3], Dariusz Bartosik[1], Lukasz Dziewit[1*]

*** Correspondence:** Dr. Lukasz Dziewit: ldziewit@biol.uw.edu.pl

TABLE S7. Toxin-antitoxin systems identified within *Polaromonas* plasmids.

Plasmid	Toxin gene	Antitoxin gene	Toxin family	Antitoxin family	Homologous TA system (found based on best BLASTP hit to the toxin protein)
pE3SP1	*pE3SP1_p010*	*pE3SP1_p009*	HigB	HigA	CFE43_21525-CFE43_21520 of *Burkholderiales* bacterium isolate PBB3 NODE_6924 [GenBank: NKIK01000079]
	pE3SP1_p036	*pE3SP1_p037*	ParE	N/A	WS67_11505-WS67_11510 of *Burkholderia singularis* TSV85 [GenBank: LOWA01000030]
	pE3SP1_p038	*pE3SP1_p039*	BrnT	BrnA	NS337_17120- NS337_17115 of *Pseudomonas psychrotolerans* NS337 [GenBank: LDSR01000038]
	pE3SP1_p047	*pE3SP1_p046*	RelE/StbE	RelB/DinJ	B7Y54_12295-B7Y54_12300 of *Polaromonas* sp. 35-63-240 [GenBank: NCFW01000268]
	pE3SP1_p048 (truncated)	*pE3SP1_p49*	ParE	N/A	SCD_n02373-SCD_n02374 of *Sulfuricella denitrificans* skB26 [GenBank: AP013066]
pE5SP1	*pE5SP1_p009*	*pE5SP1_p008*	HigB	HigA	JAB2_55680-JAB2_55670 of Janthinobacterium sp. HH100 [GenBank: LRHY01000143]
	pE5SP1_p030	*pE5SP1_p031*	ParE	N/A	WS67_11505-WS67_11510 of *Burkholderia singularis* TSV85 [GenBank: LOWA01000030]
	*pE5SP1_p035-036**	*pE5SP1_p037*	BrnT	BrnA	NS337_17120-NS337_17115 of *Pseudomonas psychrotolerans* NS337 [GenBank: LDSR01000038]
	pE5SP1_p052 (truncated)	*pE5SP1_p051*	BrnT	BrnA	UM91_07095-UM91_07090 of *Pseudomonas oryzihabitans* RIT370 [GenBank: JYKV01000005]
pE10SP1	*pE10SP1_p013-012**	*pE10SP1_p011*	BrnT	BrnA	NS337_17120-NS337_17115 of *Pseudomonas psychrotolerans* NS337 [GenBank: LDSR01000038]
	pE10SP1_p016	*pE10SP1_p017*	RelE/StbE	RelB/DinJ	B9Z41_14740-B9Z41_14735 of *Limnohabitans* sp. JirII-31 [GenBank: NESA01000013]
	*pE10SP1_p022-021**	*pE10SP1_p020*	BrnT	BrnA	NS337_17120- NS337_17115 of *Pseudomonas psychrotolerans* NS337 [GenBank: LDSR01000038]
	pE10SP1_p028	*pE10SP1_p027*	ParE	N/A	WS67_11505-WS67_11510 of *Burkholderia singularis* TSV85 [GenBank: LOWA01000030]
	pE10SP1_p045	*pE10SP1_p046*	HigB	HigA	JAB2_55680-JAB2_55670 of Janthinobacterium sp. HH100 [GenBank: LRHY01000143

pE19SP1	*pE19SP1_p008*	*pE19SP1_p009*	RelE/StbE	RelB/DinJ	B9Z41_14740-B9Z41_14735 of *Limnohabitans* sp. JirII-31 [GenBank: NESA01000013]
	*pE19SP1_p014-013**	*pE19SP1_p012*	BrnT	BrnA	NS337_17120-NS337_17115 of *Pseudomonas psychrotolerans* NS337 [GenBank: LDSR01000038]
	pE19SP1_p020	*pE19SP1_p019*	ParE	N/A	WS67_11505-WS67_11510 of Burkholderia singularis TSV85 [GenBank: LOWA01000030]
pH8NP2	*pH8NP2_p002*	*pH8NP2_p003*	BrnT	BrnA	CGL2_11216024-CGL2_11216025 of *Leptospirillum* sp. Group II '5-way CG' [GenBank: DS995263.1
pW10NP1	*pW10NP1_p019*	*pW10NP1_p018*	ParE	N/A	Pnap_4990-Pnap_4989 of P. naphthalenivorans CJ2 plasmid pPNAP06 [GenBank: CP000535]
pW11NP2	*pW11NP2_p037*	*pW11NP2_p038*	HipA	N/A	SAMN04489707_102559-SAMN04489707_102560 of *Acidovorax caeni* R-24608 [GenBank: FPBX01000025]

* possible frameshift

N/A – not assigned
