# Supplementary material for: Plasmids of Psychrotolerant Polaromonas spp. Isolated From Arctic and Antarctic Glaciers – Diversity and Role in Adaptation to Polar Environments
Source: Front Microbiol. 2018 Jun 18;9:1285. doi: 10.3389/fmicb.2018.01285 (PMC6015842; doi:10.3389/fmicb.2018.01285)
Supplement: Supplementary file 8 [file Table_8.PDF]

Supplementary Material

Plasmids of Psychrotolerant *Polaromonas* spp. Isolated from Arctic and Antarctic Glaciers – Diversity and Role in Adaptation to Polar Environments

Anna Ciok[1], Karol Budzik[1], Marek K. Zdanowski[2], Jan Gawor[3], Jakub Grzesiak[2], Przemyslaw Decewicz[1], Robert Gromadka[3], Dariusz Bartosik[1], Lukasz Dziewit[1*]

*** Correspondence:** Dr. Lukasz Dziewit: ldziewit@biol.uw.edu.pl

TABLE S8. Conjugal transfer systems identified within *Polaromonas* plasmids.

**Plasmid name**	**Relaxase gene (coordinates)**	**Relaxase family**	**Other *tra* genes (coordinates)**
pE5SP1	*pE5SP1_p049* (47094 – 47702) truncated gene	Not determined	Not found
pH1NP1	pH1NP1_p004 (4092 – 6230)	MOB_P	pH1NP1_p003 (3846 – 3418)
pH8NP2	pH8NP2_p012 (10484 – 13210)	MOB_{Q1}	pH8NP2_p011 (10176 – 10487)
pW5NP1	pW5NP1_p010 (7399 – 9573)	MOB_P	pW5NP1_p009 (7184 – 6690)
pW10NP1	pW10NP1_p013 (13088 – 15715)	MOB_{Q1}	pW10NP1_p012 (12690 – 13001)
