# Supplementary material for: Plasmids of Psychrotolerant Polaromonas spp. Isolated From Arctic and Antarctic Glaciers – Diversity and Role in Adaptation to Polar Environments
Source: Front Microbiol. 2018 Jun 18;9:1285. doi: 10.3389/fmicb.2018.01285 (PMC6015842; doi:10.3389/fmicb.2018.01285)
Supplement: Supplementary file 9 [file Table_9.PDF]

Supplementary Material

Plasmids of Psychrotolerant *Polaromonas* spp. Isolated from Arctic and Antarctic Glaciers – Diversity and Role in Adaptation to Polar Environments

Anna Ciok[1], Karol Budzik[1], Marek K. Zdanowski[2], Jan Gawor[3], Jakub Grzesiak[2], Przemyslaw Decewicz[1], Robert Gromadka[3], Dariusz Bartosik[1], Lukasz Dziewit[1*]

*** Correspondence:** Dr. Lukasz Dziewit: ldziewit@biol.uw.edu.pl

TABLE S9. Summary of phenotypic modules found within analysed *Polaromonas* plasmids.

Plasmid name	Protein	Predicted protein function [Enzyme commission number]
Protection against oxidative stress		
pE3SP1	pE3SP1_p014,<br>pE3SP1_p066	lipoate synthase, COG0320 [EC: 2.8.1.8]
pE3SP1	pE3SP1_p018,<br>pE3SP1_p071	glutaredoxin-related protein, COG0278
pE3SP1	pE3SP1_p025<br>pE3SP1_p024<br>pE3SP1_p023<br>pE3SP1_p022<br>pE3SP1_p021<br>pE3SP1_p020<br>pE3SP1_p017<br>pE3SP1_p015	Fe-S cluster assembly iron-binding protein IscA, COG0316<br>Fe-S cluster assembly scaffold protein SufB, COG0719<br>Fe-S cluster assembly ATPase SufC, COG0396<br>Fe-S cluster assembly protein SufD, COG0719<br>cysteine sulfinate desulfinase SufS, COG0520 [EC: 2.8.1.7/4.4.1.16]<br>Fe-S cluster assembly protein IscU, COG0822<br>Fe-S cluster biogenesis scaffold protein Nfu/NifU, COG0694<br>Fe-S cluster biogenesis scaffold protein Nfu/NifU, COG0694
pE3SP1	pE3SP1_p079<br>pE3SP1_p078<br>pE3SP1_p077<br>pE3SP1_p076<br>pE3SP1_p075<br>pE3SP1_p074<br>pE3SP1_p073<br>pE3SP1_p070<br>pE3SP1_p063	Fe-S cluster assembly iron-binding protein IscA, COG0316<br>Fe-S cluster assembly iron-binding protein IscA, COG0316<br>Fe-S cluster assembly scaffold protein SufB, COG0719<br>Fe-S cluster assembly ATPase SufC, COG0396<br>Fe-S cluster assembly protein SufD, COG0719<br>cysteine sulfinate desulfinase SufS, COG0520 [EC: 2.8.1.7/4.4.1.16]<br>Fe-S cluster assembly protein IscU, COG0822<br>Fe-S cluster biogenesis scaffold protein Nfu/NifU, COG0694<br>Fe-S cluster biogenesis scaffold protein Nfu/NifU, COG0694
pE5SP1	pE5SP1_p014	lipoate synthase, COG0320 [EC: 2.8.1.8]
pE5SP1	pE5SP1_p018	glutaredoxin-related protein, COG0278
pE5SP1	pE5SP1_p025<br>pE5SP1_p024<br>pE5SP1_p023<br>pE5SP1_p022<br>pE5SP1_p021<br>pE5SP1_p020<br>pE5SP1_p017<br>pE5SP1_p015	Fe-S cluster assembly iron-binding protein IscA, COG0316<br>Fe-S cluster assembly scaffold protein SufB, COG0719<br>Fe-S cluster assembly ATPase SufC, COG0396<br>Fe-S cluster assembly protein SufD, COG0719<br>cysteine sulfinate desulfinase SufS, COG0520 [EC: 2.8.1.7/4.4.1.16]<br>Fe-S cluster assembly protein IscU, COG0822<br>Fe-S cluster biogenesis scaffold protein Nfu/NifU, COG0694<br>Fe-S cluster biogenesis scaffold protein Nfu/NifU, COG0694

pE10SP1	pE10SP1_p032<br>pE10SP1_p033<br>pE10SP1_p034<br>pE10SP1_p035<br>pE10SP1_p036<br>pE10SP1_p037<br>pE10SP1_p040<br>pE10SP1_p042	Fe-S cluster assembly iron-binding protein IscA, COG0316<br>Fe-S cluster assembly scaffold protein SufB, COG0719<br>Fe-S cluster assembly ATPase SufC, COG0396<br>Fe-S cluster assembly protein SufD, COG0719<br>cysteine sulfinate desulfinase SufS, COG0520 [EC: 2.8.1.7/4.4.1.16]<br>Fe-S cluster assembly protein IscU, COG0822<br>Fe-S cluster biogenesis scaffold protein Nfu/NifU, COG0694<br>Fe-S cluster biogenesis scaffold protein Nfu/NifU, COG0694
pE10SP1	pE10SP1_p039	glutaredoxin-related protein, COG0278
pE10SP1	pE10SP1_p043	lipoate synthase, COG0320 [EC: 2.8.1.8]
pH6NP1	pH6NP1_p028	catalase-peroxidase, COG0376 [EC: 1.11.1.21]
pH6NP1	pH6NP1_p063	catalase, COG0753 [EC: 1.11.1.6]
pW11NP2	pW11NP2_p035	glutathione S-transferase, COG0625 [EC: 2.5.1.18]
Protection against UV radiation		
pH6NP1	pH6NP1_p004	DNA polymerase V, subunit UmuC, COG0389
Protection against low temperatures		
pW11NP2	pW11NP2_p017	fatty acyl-CoA synthetase, COG0318 [EC: 6.2.1.44]
Uptake and utilisation of various organic compounds		
pE3SP1	pE3SP1_p041	periplasmic DMSO/TMAO reductase, COG2041
pE3SP1	pE3SP1_p043	glucose/arabinose dehydrogenase, COG2133
pE3SP1	pE3SP1_p057	acetolactate synthase, small subunit IlvH, COG0440 [EC: 2.2.1.6]
pE3SP1	pE3SP1_p058	acetolactate synthase, large subunit IlvB, COG0028 [EC: 2.2.1.6]
pE3SP1	pE3SP1_p065	rhodanese-related sulfurtransferase, COG0607
pE3SP1	pE3SP1_p068	GTP cyclohydrolase I, COG0302 [EC: 3.5.4.16]
pE3SP1	pE3SP1_p085	aconitate hydratase [EC: 4.2.1.3]
pE5SP1	pE5SP1_p039	glucose/arabinose dehydrogenase, COG2133
pE10SP1	pE10SP1_p018	glucose/arabinose dehydrogenase, COG2133
pE10SP1	pE10SP1_p057<br>pE10SP1_p058<br>pE10SP1_p059	ABC-type amino acid transport system, periplasmic component, COG0834<br>ABC-type amino acid transport system, permease component, COG0765<br>ABC-type amino acid transport system, ATPase component, COG1126 [EC: 3.6.3.-]
pE10SP1	pE10SP1_p060	selenocysteine lyase, COG0520
pE10SP1	pE10SP1_p061	dihydroorotase or related cyclic amidohydrolase, COG0044 [EC: 3.5.2.2]
pE10SP1	pE10SP1_p063	ureidoglycolate lyase, COG3194 [EC: 4.3.2.3]
pE10SP1	pE10SP1_p064	cyanuric acid hydrolase [EC: 3.5.2.15]
pE10SP1	pE10SP1_p065<br>pE10SP1_p066<br>pE10SP1_p067<br>pE10SP1_p068	ATPase component of ABC-type Fe3+/spermidine/putrescine transport system, COG3842 [EC: 3.6.3.31]<br>spermidine/putrescine-binding periplasmic protein, COG0687<br>ABC-type spermidine/putrescine transport system, permease component I, COG1176<br>ABC-type spermidine/putrescine transport system, permease component II, COG1177
pE10SP1	pE10SP1_p069	Asp/Glu/hydantoin racemase, COG4126
pE10SP1	pE10SP1_p070	putative polysaccharide deacetylase
pE10SP1	pE10SP1_p079	aconitate hydratase [EC: 4.2.1.3]
pE19SP1	pE19SP1_p010	glucose/arabinose dehydrogenase, COG2133
pH6NP1	pH6NP1_p056	pimeloyl-ACP methyl ester carboxylesterase, COG0596
pH6NP1	pH6NP1_p057	acetyl esterase/lipase, COG0657
pW11NP2	pW11NP2_p012	glucose/arabinose dehydrogenase, COG2133
pW11NP2	pW11NP2_p016	periplasmic component of ABC-type branched-chain amino acid transport system, COG0683

Transport of metal ions/Resistance to heavy metals		
pE3SP1	pE3SP1_p089	P-type heavy metal-transporting ATPase, ZntA, COG2217 [EC: 3.6.3.54]
pE5SP1	pE5SP1_p033	TonB-dependent outer membrane receptor protein, COG1629
pE5SP1	pE5SP1_p034	Zn-binding component of ABC-type Zn uptake system, ZnuA, COG0803
pE10SP1	pE10SP1_p023	Zn-binding component of ABC-type Zn uptake system, ZnuA, COG0803
pE10SP1	pE10SP1_p025	TonB-dependent outer membrane receptor protein, COG1629
pE10SP1	pE10SP1_p054	P-type heavy metal-transporting ATPase, ZntA, COG2217 [EC: 3.6.3.54]
pE19SP1	pE19SP1_p015	Zn-binding component of ABC-type Zn uptake system, ZnuA, COG0803
pE19SP1	pE19SP1_p017	TonB-dependent outer membrane receptor protein, COG1629
pH6NP1	pH6NP1_p040<br>pH6NP1_p041<br>pH6NP1_p042<br>pH6NP1_p043<br>pH6NP1_p044	DNA-binding transcriptional regulator, MerR family, COG0789<br>mercuric transport protein, integral membrane component, MerT<br>mercuric transport protein, periplasmic component, MerP<br>mercuric transport protein, MerC<br>mercuric reductase, MerA [EC: 1.16.1.1]
pH6NP1	pH6NP1_p047	divalent metal cation transporter, CDF family, COG0053
pH6NP1	pH6NP1_p054	MntH transporter, COG1914
