# Supplementary material for: Plasmids of Psychrotolerant Polaromonas spp. Isolated From Arctic and Antarctic Glaciers – Diversity and Role in Adaptation to Polar Environments
Source: Front Microbiol. 2018 Jun 18;9:1285. doi: 10.3389/fmicb.2018.01285 (PMC6015842; doi:10.3389/fmicb.2018.01285)
Supplement: Supplementary file 10 [file Table_10.PDF]

Supplementary Material

Plasmids of Psychrotolerant *Polaromonas* spp. Isolated from Arctic and Antarctic Glaciers – Diversity and Role in Adaptation to Polar Environments

Anna Ciok[1], Karol Budzik[1], Marek K. Zdanowski[2], Jan Gawor[3], Jakub Grzesiak[2], Przemyslaw Decewicz[1], Robert Gromadka[3], Dariusz Bartosik[1], Lukasz Dziewit[1*]

*** Correspondence:** Dr. Lukasz Dziewit: ldziewit@biol.uw.edu.pl

TABLE S10. MIC values [mM] for heavy metal ions tested in heterologous hosts.

Strain		*A. tumefacies* LBA288				*E. coli* DH5α				*P. aeruginosa* PAO1161				*V. paradoxus* EPS			
Temperature		15°C		30°C		15°C		37°C		15°C		37°C		15°C		30°C	
Resistance module	Metal ion	Control[#]	Module*	Control	Module	Control	Module	Control	Module	Control	Module	Control	Module	Control	Module	Control	Module
CDF-H6N	Cd^{2+}	1	1	1	1	1	1	1	1	1	1	2	2	1	1	1	1
	Co^{2+}	1	1	2	2	2	2	4	4	1	1	2	2	1	1	1	1
	Cu^{2+}	5	5	5	5	5	5	5	5	6	6	7	7	4	4	4	4
	Mn^{2+}	20	20	20	20	20	20	20	20	**3**	**2**	**5**	**4**	3	3	3	3
	Ni^{2+}	6	6	6	6	5	5	5	5	2	2	3	3	2	2	2	2
	Zn^{2+}	4	4	4	4	2	2	2	2	8	8	10	10	**3**	**4**	**3**	**4**
MER-H6N	Hg^{2+}	**0.05**	**0.1**	**0.05**	**0.1**	0.05	0.05	0.05	0.05	**0.15**	**0.05**	**0.15**	**0.05**	0.05	0.05	0.05	0.05
ZNT-E3S	Cd^{2+}	1	1	1	1	1	1	1	1	1	1	2	2	1	1	1	1
	Co^{2+}	1	1	2	2	2	2	4	4	1	1	2	2	1	1	1	1
	Cu^{2+}	5	5	5	5	5	5	5	5	6	6	7	7	4	4	4	4
	Hg^{2+}	0.05	0.05	0.05	0.05	0.05	0.05	0.05	0.05	0.15	0.15	0.15	0.15	0.05	0.05	0.05	0.05
	Ni^{2+}	6	6	6	6	5	5	5	5	2	2	3	3	2	2	2	2
	Zn^{2+}	4	4	4	4	2	2	2	2	8	8	10	10	**3**	**4**	**3**	**5**
ZNT-E10S	Cd^{2+}	1	1	1	1	1	1	1	1	1	1	2	2	1	1	1	1
	Co^{2+}	1	1	2	2	2	2	4	4	1	1	2	2	1	1	1	1
	Cu^{2+}	5	5	5	5	5	5	5	5	6	6	7	7	4	4	4	4
	Hg^{2+}	0.05	0.05	0.05	0.05	0.05	0.05	0.05	0.05	0.15	0.15	0.15	0.15	0.05	0.05	0.05	0.05
	Ni^{2+}	6	6	6	6	5	5	5	5	2	2	3	3	2	2	2	2
	Zn^{2+}	4	4	4	4	2	2	2	2	8	8	10	10	**3**	**4**	**3**	**5**
ZNU-E5S	Zn^{2+}	4	4	4	4	2	2	2	2	8	8	10	10	3	3	3	3
ZNU-E19S	Zn^{2+}	4	4	4	4	2	2	2	2	8	8	10	10	3	3	3	3

Changes in MIC values between the control and tested genetic module are shown in bold

[#] Control – strain carrying “empty” plasmid pBBR1 MCS-2

* Module – strains carrying pBBR1 MCS-2 derivatives with cloned predicted resistance modules: CDF-H6N – predicted cadmium, cobalt, copper, manganese, nickel and/or zinc resistance module of plasmid pH6NP1; MER-H6N – predicted mercury resistance module of plasmid pH6NP1; ZNT-E3S and ZNT-E10S – predicted cadmium, cobalt, copper, mercury, nickel and/or zinc resistance modules of plasmids pE3SP1 and pE10SP1, respectively; ZNU-E5S and ZNU-E19S – predicted zinc resistance module of plasmids pE5SP1 and pE19SP1, respectively.
